# A Systematic Review of Recently Reported Marine Derived Natural Product Kinase Inhibitors

**DOI:** 10.3390/md17090493

**Published:** 2019-08-23

**Authors:** Te Li, Ning Wang, Ting Zhang, Bin Zhang, Thavarool P. Sajeevan, Valsamma Joseph, Lorene Armstrong, Shan He, Xiaojun Yan, C. Benjamin Naman

**Affiliations:** 1 Li Dak Sum Yip Yio Chin Kenneth Li Marine Biopharmaceutical Research Center, Department of Marine Pharmacy, College of Food and Pharmaceutical Sciences, Ningbo University, Ningbo 315800, Zhejiang, China; 2 Institute of Drug Discovery Technology, Ningbo University, Ningbo 315211, Zhejiang, China; 3 National Centre for Aquatic Animal Health, Cochin University of Science and Technology, Fine Arts Avenue, Kochi, Kerala 682016, India; 4 Department of Pharmaceutical Sciences, State University of Ponta Grossa, Ponta Grossa, Paraná 84030900, Brazil; 5 Center for Marine Biotechnology and Biomedicine, Scripps Institution of Oceanography, University of California, San Diego, La Jolla, CA 92093, USA

**Keywords:** kinase inhibitors, drug development, marine natural products

## Abstract

Protein kinases are validated drug targets for a number of therapeutic areas, as kinase deregulation is known to play an essential role in many disease states. Many investigated protein kinase inhibitors are natural product small molecules or their derivatives. Many marine-derived natural products from various marine sources, such as bacteria and cyanobacteria, fungi, animals, algae, soft corals, sponges, etc. have been found to have potent kinase inhibitory activity, or desirable pharmacophores for further development. This review covers the new compounds reported from the beginning of 2014 through the middle of 2019 as having been isolated from marine organisms and having potential therapeutic applications due to kinase inhibitory and associated bioactivities. Moreover, some existing clinical drugs based on marine-derived natural product scaffolds are also discussed.

## 1. Introduction

Oceans and seas occupy almost 70% of the Earth’s surface, and are estimated to host about 80% of all living species [1]. The rich biodiversity of the marine environment has been shown to yield equally rich chemical diversity of marine natural products isolated from the different organisms that have been studied since the 1940s [2]. In the same time period, roughly two-thirds of small molecules drugs with FDA or equivalent regulatory agency approvals have been granted for natural products or derivatives thereof [3]. This has certainly included many exceptionally impactful antibiotic or antiparasitic drugs and oncology therapeutics, among others [4,5,6,7]. Indeed, the published structures of natural products appear to correlate well with the chemical space occupied by approved drugs, and the natural product drug discovery continues to yield new and interesting compounds [2,8,9]. According to the MarinLit database (http://pubs.rsc.org/marinlit), more than 28,000 marine natural products have been reported after being isolated from a variety of marine sources; such as algae, ascidians, bryozoa, corals, microorganisms, sea hares, sea squirts, sponges, etc. [10]. The known and yet-to-be discovered marine natural product compounds represent a vast natural resource library. In the golden age of natural product research, many marine-derived scaffolds have been applied to clinical drug discovery and development due to new ideas and breakthroughs in screening technologies: (a) The mesophotic zone is increasingly recognized as being valuable for the discovery of new drug structures and unique activities [11]; (b) Computational methodologies play an essential role in the exploration of the biological activity and molecular structural networking of marine natural products [12]; (c) Databases of marine natural products (e.g. MarinLit: http://pubs.rsc.org/marinlit/ and Dictionary of Marine Natural Products: http://dmnp.chemnetbase.com/) are available to facilitate dereplication and discovery efforts and improve marine natural products research; (d) Chemical synthesis of complex molecules has become increasingly feasible at even gram-scale quantities that allow for the further biological interrogation and drug development of natural products previously isolated in low yields [13].

**Figure 1:**
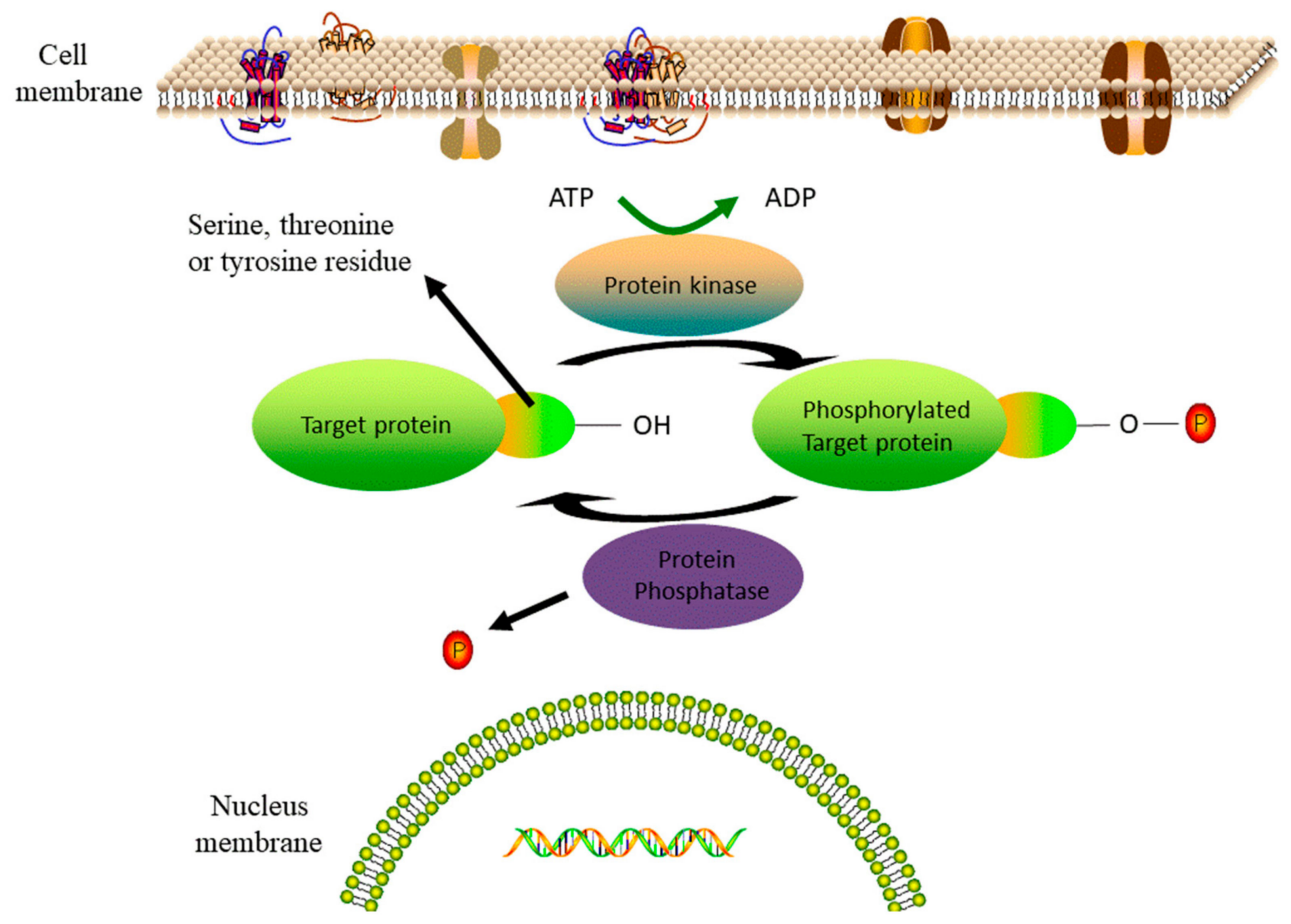
The catalytic cycle for protein phosphorylation by a protein kinase. Red circles represent phosphate groups. Adapted from content in Ref [16].

Most of the kinase inhibitor drugs approved thus far are adenosine triphosphate (ATP) competitive inhibitors that have various practical off-target liabilities. Four major classes of kinases exist in mammalian signaling pathways, and these can be classified broadly by substrate specificity: Serine/threonine kinases, tyrosine kinases, dual kinases (both Ser/Thr and Tyr), and lipid kinases [14]. Protein kinases share a common mechanism that is demonstrated in Figure 1, in which the protein function is activated or inactivated by the transfer of a phosphate group from ATP to the free hydroxy of a serine, threonine or tyrosine on the targeted protein, whereas protein phosphatases remove a phosphate group from phosphorylated amino acids and thereby effectively reverse the effect [15,16,17,18]. There are 38 FDA-approved small molecule kinase inhibitors available for the treatment of different diseases, such as cancers, immunological, inflammatory, degenerative, metabolic, cardiovascular and infectious diseases [19,20], and many more candidates still in clinical development. Some new biological therapeutics, in the way of monoclonal antibodies, have been discovered or engineered to successfully target some kinases [21,22]. A major challenge in the development of new kinase inhibitors is to overcome the toxicity or non-specificity of the existing drugs that act as ATP binding site competitors. Since small-molecule natural products are produced by and interact with proteins in their natural settings, some are known to function as signaling molecules among many forms of life and have been creatively repurposed for human health applications. Accordingly, some marine natural products have served as drug lead compounds, and these provide an abundant resource for the discovery of next-generation kinase inhibitors that target allosteric regions away from the ATP binding sites [23,24,25,26] or by stabilizing inactive conformations to prevent the function of certain kinases [27,28,29,30]. This is particularly relevant for the treatment of cancers and bacterial infections.

This review provides insight into the kinase inhibitors isolated from marine sources (bacteria and cyanobacteria, fungi, animals, algae, soft corals and sponges) that have been reported in the last five years since 2014 and highlight the associated biological activities and potential clinical applications. Examples of successful applications of marine natural products lead compound discovery and use of new drug screening technologies that have enabled rapid expansion in the field are accordingly provided.

## 2. Discussion

### 2.1. New Kinase Inhibitors Discovered from Marine Organisms

**Figure 2:**
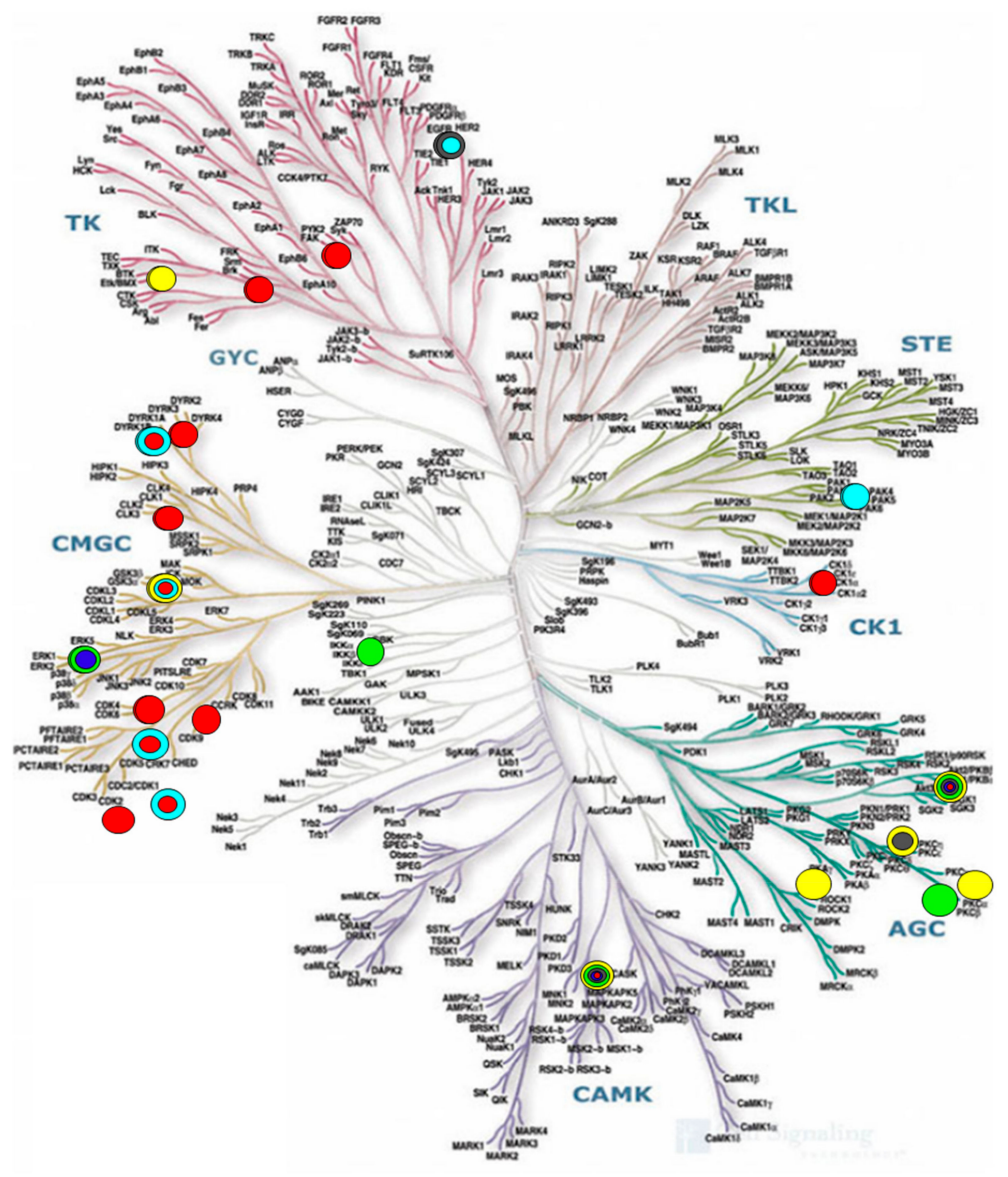
Availability of some marine-derived kinase inhibitors with activity on the phylogenetic tree of the human protein kinase family. Color codes indicate the producing or source organisms. Yellow: Marine bacteria. Green: Marine fungi. Gray: Marine soft coral. Light blue: Marine animals. Dark blue: Marine algae. Red: Marine sponges. Adapted from refs [31] and [32] with permission, the original illustration reproduced courtesy of Cell Signaling Technology, Inc. (www.cellsignal.com), 2019.

Marine-derived natural products include a wide range of molecular classes such as alkaloids, macrolides, polypeptides, and terpenoids, and have different and interesting structures that play an important role in biological activities and clinical drug applications. As it pertains to the kinase research, a number of marine-derived kinase inhibitors have come from many different sources and target many different protein kinases (Figure 2). In the last five years covered in this review, from 2014–2019, there have been many results reported in the kinase research, and accordingly new biologically active kinase inhibitors that were discovered from various ocean life forms that include bacteria and cyanobacteria, fungi, animals, algae, soft corals and sponges. The initial reports of these natural product small molecules most typically include some rudimentary bioactivity test data, and further research can or does go on to explore more deeply the pharmacological potential of each. The new natural products reported during the period of this review are described systematically in the following sub-sections, divided by the source organisms reported for each.

#### 2.1.1. Kinase Inhibitors from Marine Bacteria

**Figure 3:**
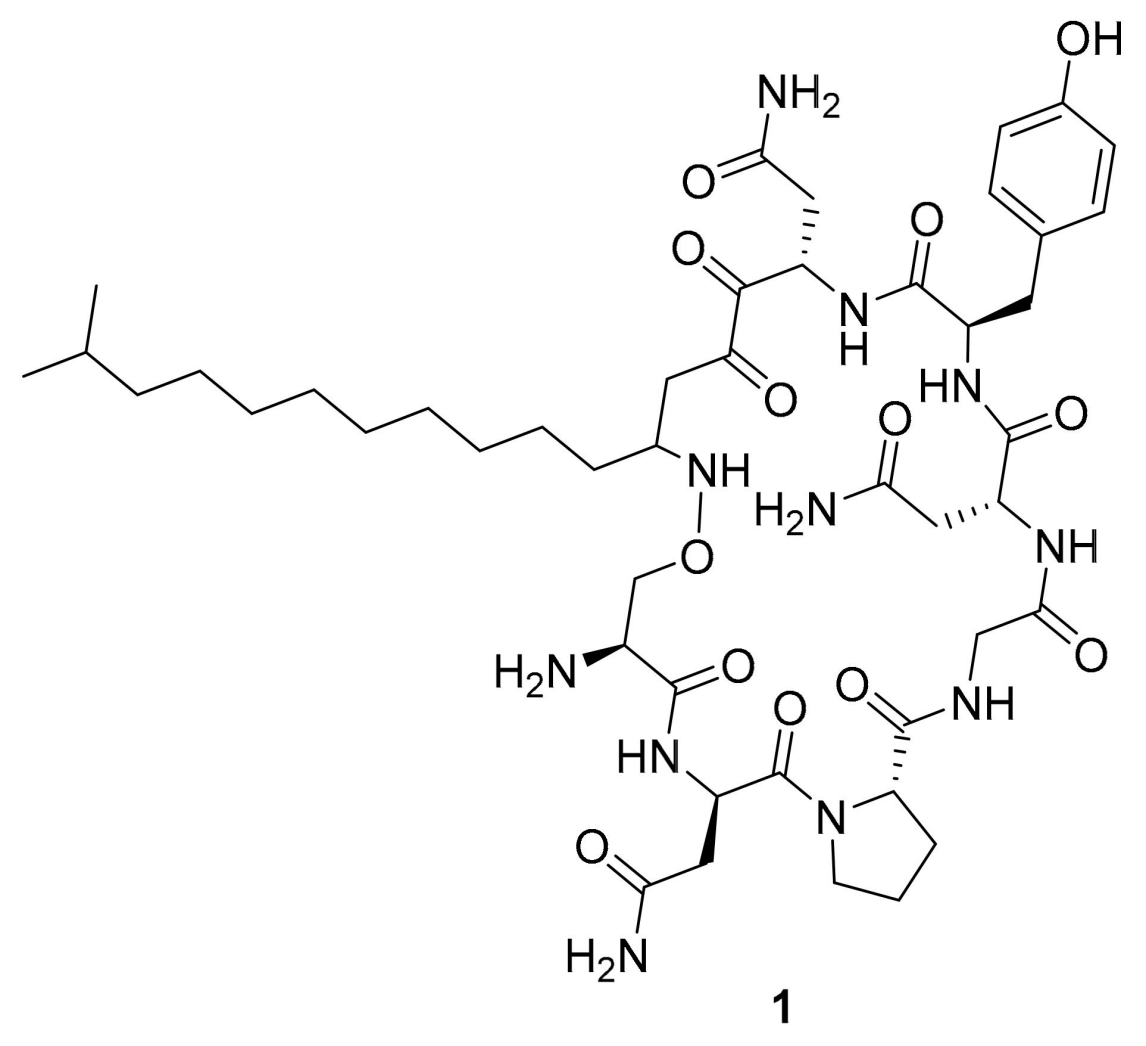
Structure of the lipopeptide iturin A (**1**).

**Figure 4:**
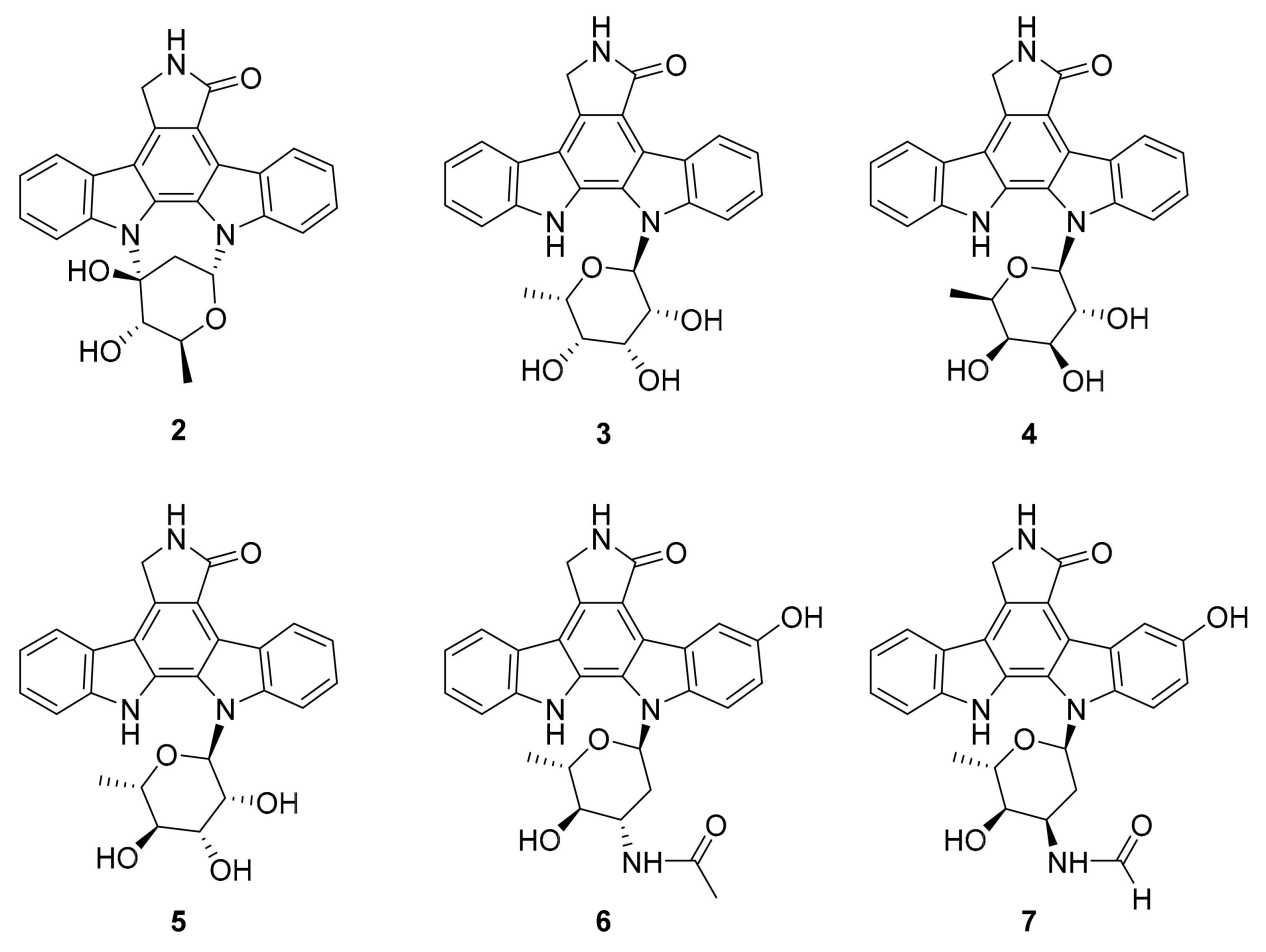
Structures of indolocarbazole alkaloids **2**–**7**.

**Figure 5:**
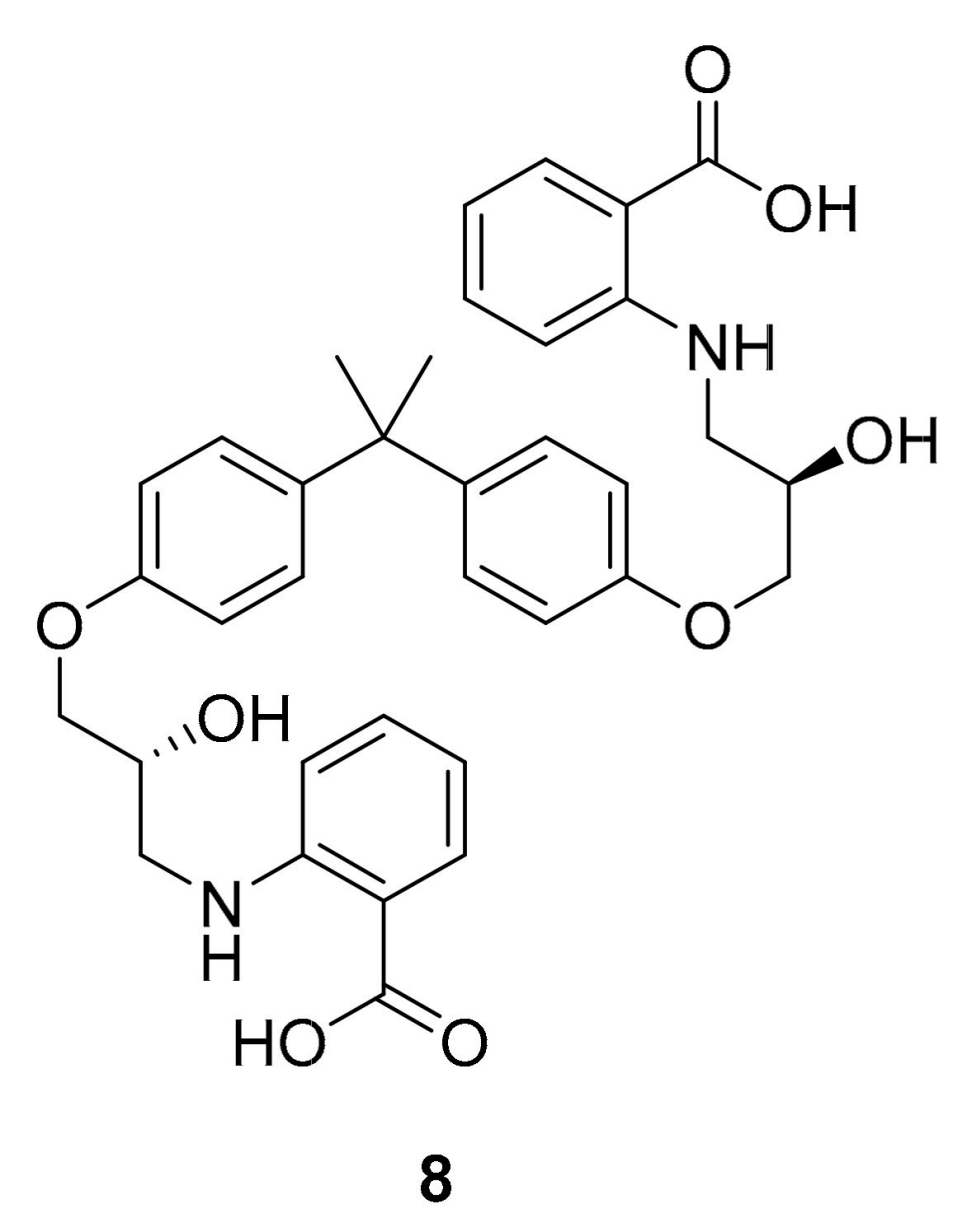
Structure of compound **8**.

**Figure 6:**
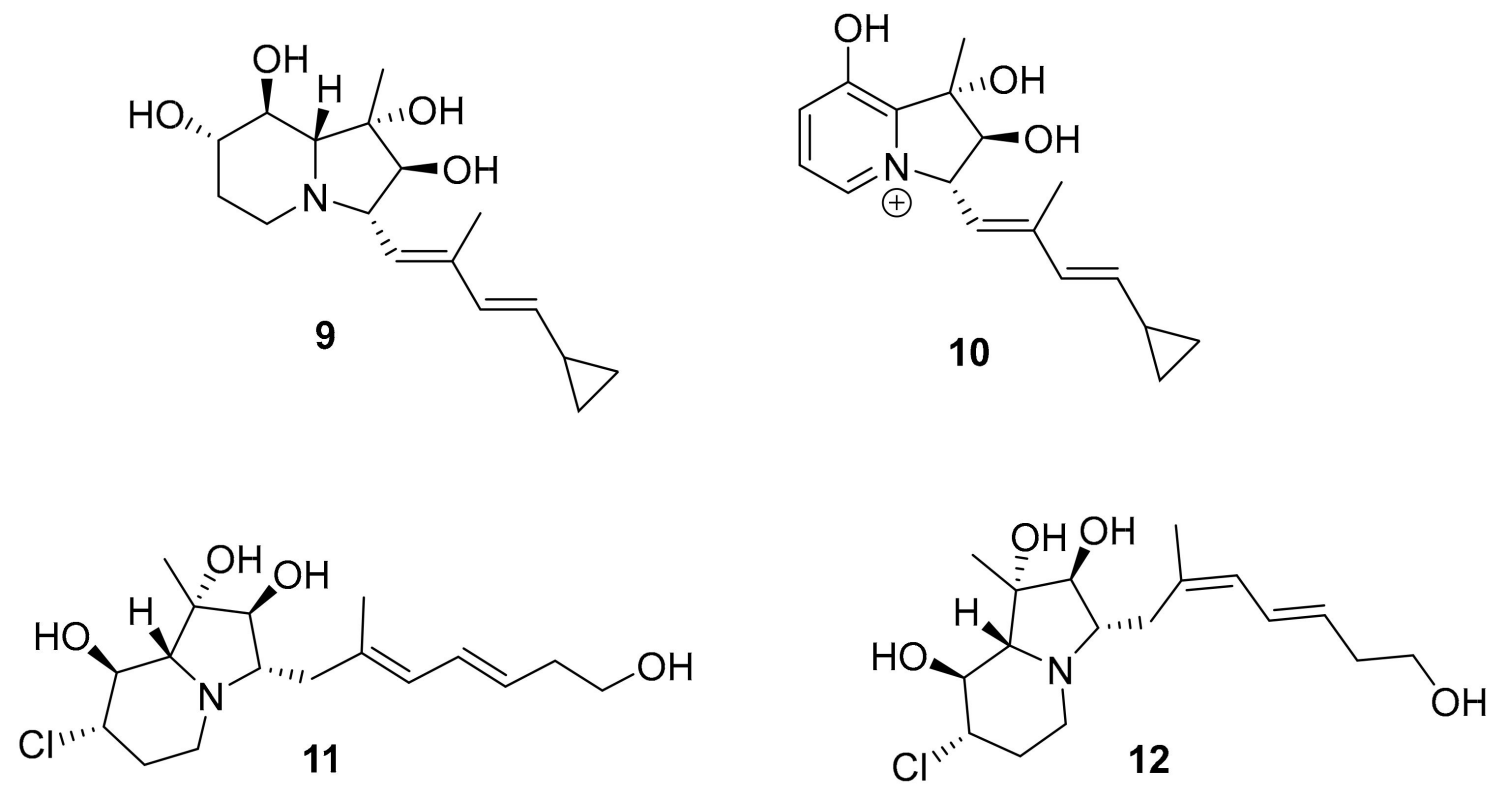
Structures of cyclizidine-type alkaloids **9**–**12**.

**Figure 7:**
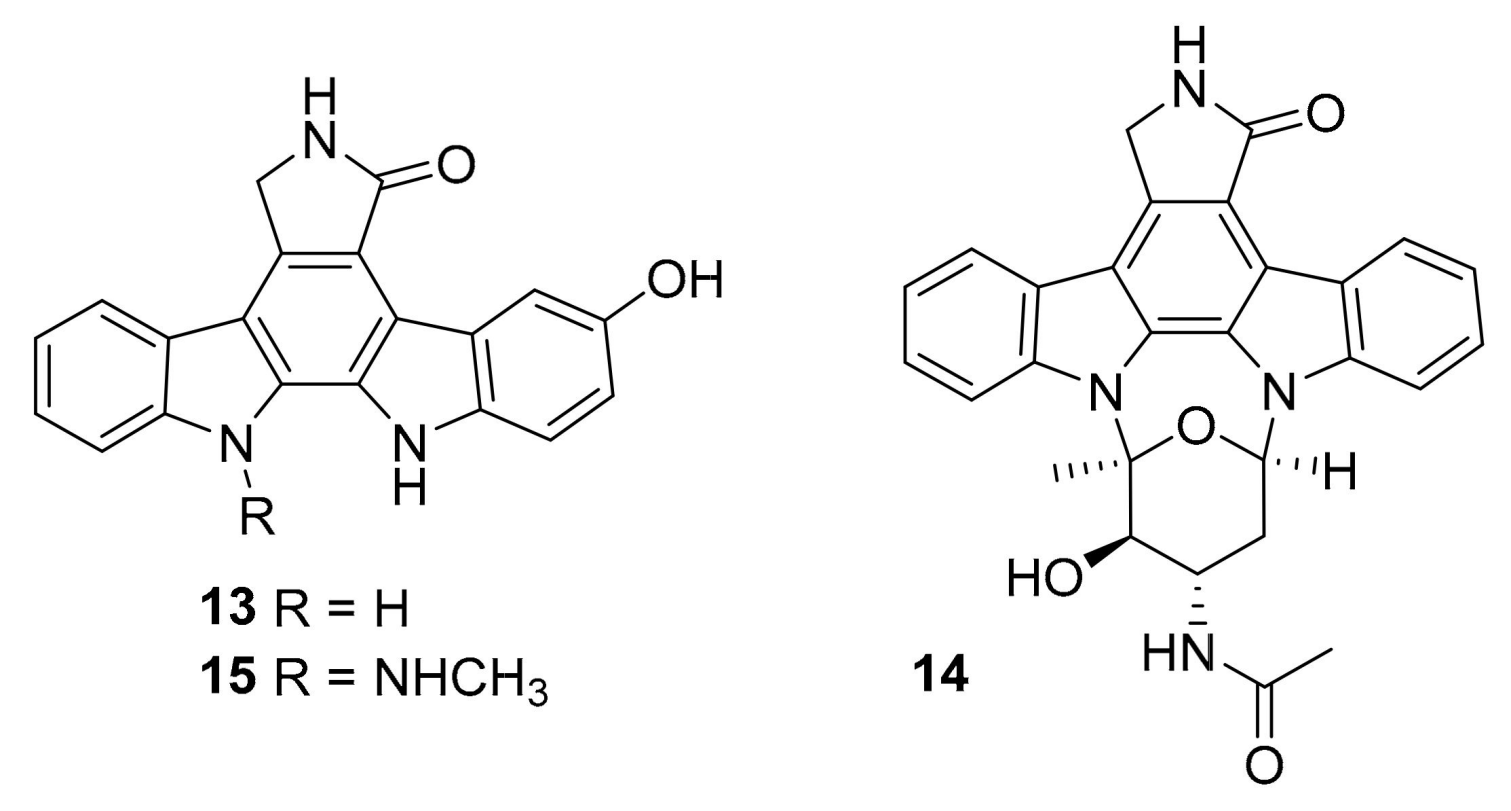
Structure of indolocarbazole alkaloids **13**–**15**.

**Figure 8:**
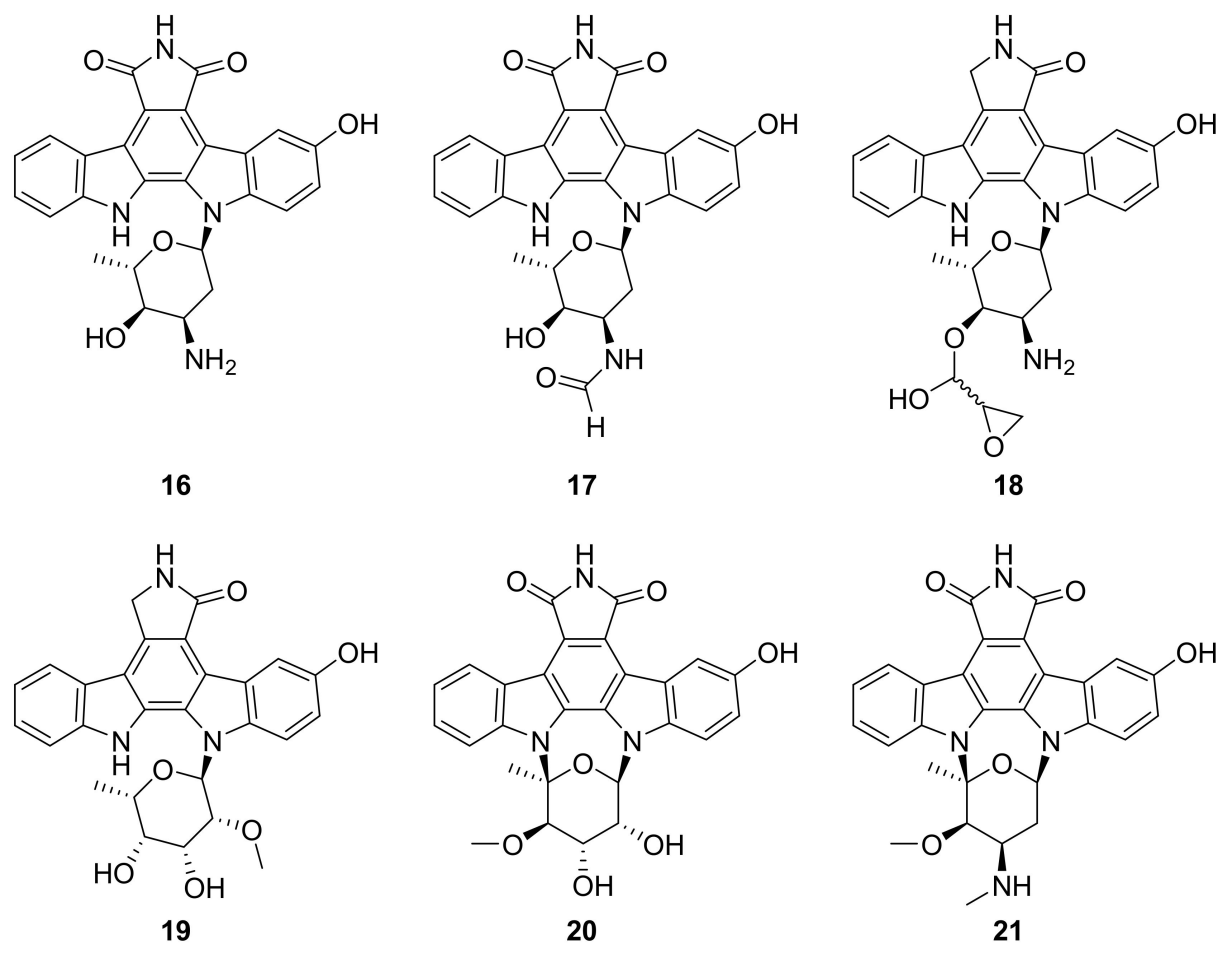
Structures of staurosporine derivatives **16**–**21**.

**Figure 9:**
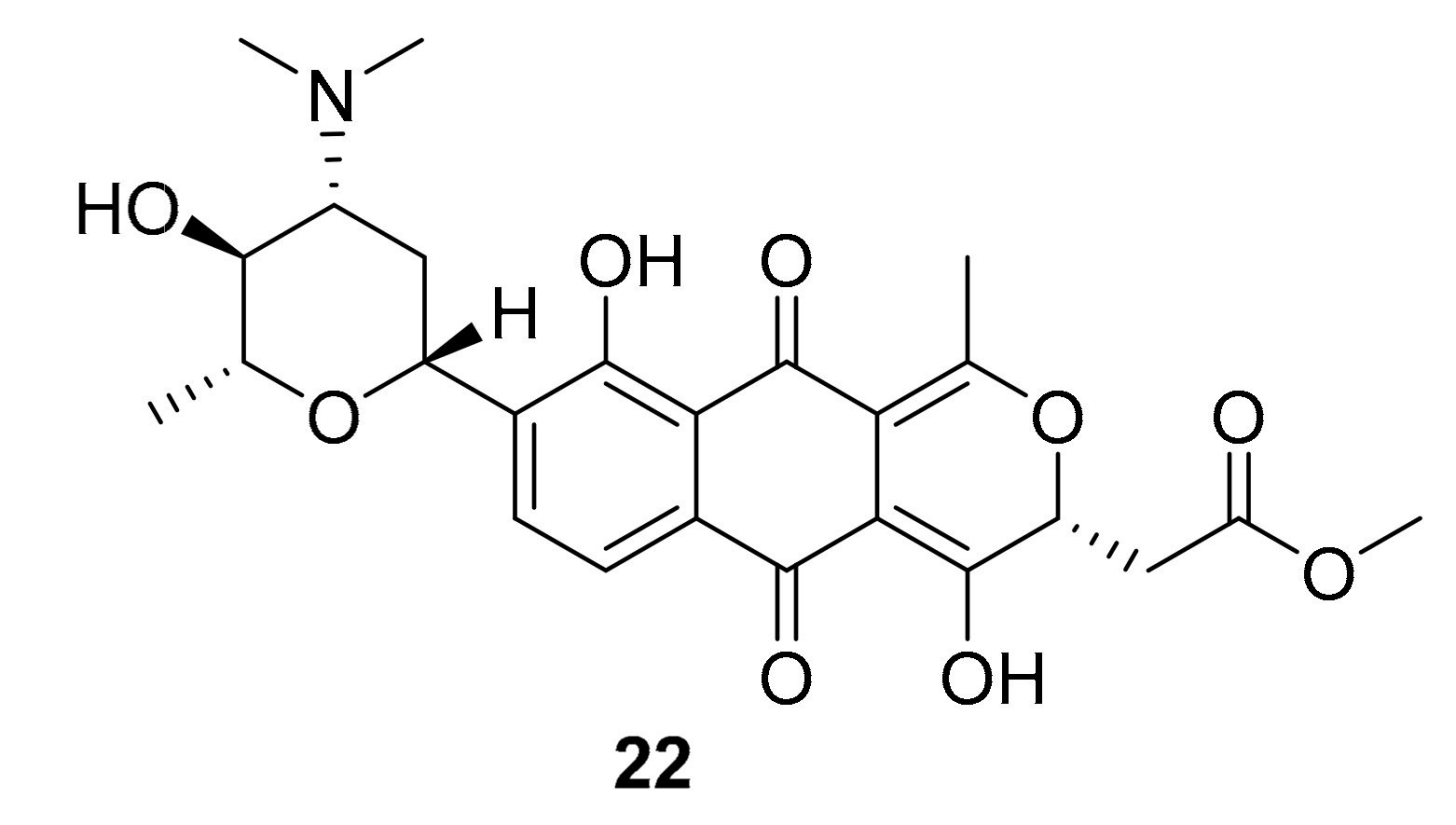
Structure of compound **22**.

In the time period covered in this review, 22 new natural product kinase inhibitors (**1–22**) were reported after being isolated from marine bacteria. These are listed in Table 1 and shown by structure in Figure 3, Figure 4, Figure 5, Figure 6, Figure 7, Figure 8 and Figure 9. The specific kinases reportedly involved in the biological activities of these compounds are described in the following paragraphs. Since staurosporine and its derivatives have been isolated and reported from marine bacteria, these are considered for the purpose of this review as being marine-derived natural products even though staurosporine itself and other analogues are also produced by terrestrial organisms. These are accordingly summarized in this section, for basic discoveries, and also in Section 2.2, for the preclinical and clinical candidates.

In 2015, Goutam Dey et al. reported the anticancer activity of a lipopeptide, iturin A (**1**, Figure 3), which was isolated from the marine bacterium *Bacillus megaterium* [33]. In the same report, compound **1** was shown to inhibit p-Akt kinase, p-MAPK and p-GSK3β [33]. In other investigations, iturin A has been found to induce antiproliferative and apoptotic effects in breast cancer cells in vitro (IC_50_ values of MDA-MB-231, MCF-7,MDA-MB-468 and T47D cells were 7.98 ± 0.19, 12.16 ± 0.24, 13.30 ± 0.97 and 26.29 ± 0.78 μM, respectively) and in vivo [33]. These results suggest that iturin A, with its mechanism of Akt inhibition, may be useful for the development of a drug for this or other types of cancers. In 2018, Biao Zhou et al. reported the isolation of three new indolocarbazole alkaloids (**2**–**4**) along with nine known compounds (including **5**) from the marine bacterium *Streptomyces* sp. A65 [34]. Compounds **3**–**5** (Figure 4) inhibited PKC and BTK (IC_50_ between 0.25 μM and 1.91 μM), and compound **2** was not active [34]. The structural similarities of **2**–**5** along with the observed biological activities that include IC_50_ values across two or more orders of magnitude show the beginnings of a natural structural activity relationship (SAR). It would be interesting if this could be expanded by accessing further analogues through targeted isolation studies or synthetic chemistry.

In 2018, Le-Le Qin et al. reported two new indolocabazole analogues, compounds **6** and **7** (Figure 4) along with a related compound (**8**, Figure 5), isolated from a marine bacterium *Streptomyces* sp. A68 [35]. These compounds inhibited PKC-α, ROCK II and BTK with IC_50_ values ranging from 0.17 to 3.24 μM, and had cytotoxic activity in vitro against PC3 human prostate cancer cells [35]. The indolocarbazole alkaloid scaffold represented in these molecules has also been popular for the development of kinase inhibitory drugs. Comparing compounds **2**–**7**, it can be considered that the position of the oxygen atom on the tetrahydrofuran subunit and its para-hydroxy and carbamic acid moieties may have important effects on the kinase inhibitor activity of each molecule. 

In 2018, Yong-Jun Jiang et al. reported eight new and one known cyclizidine-type alkaloids including **9–12** (Figure 6) from the marine-derived actinomycete *Streptomyces* sp. HNA39 [36]. This class of molecules was first discovered only in 1982 from *Streptomyces* sp. NCIB 11649 [37,38,39]. From this group, compound **9** inhibited the in vitro survival of PC3 human prostate (IC_50_ = 0.52 ± 0.03 μM) and HCT116 human colorectal (IC_50_ = 8.3 ± 0.1 μM) cancer cells [36]. Compound **9** has four hydroxy groups on the indolizine core subunit, which may be important for producing the selectivity observed, since the mechanism of action is likely related to the Michael acceptor on the side chain. Furthermore, compounds **9**–**12** had some moderate inhibition against the ROCK2 (IC_50_ 7.0 ± 0.8 to 42 ± 3 μM) [36]. 

In 2018, Jia-Nan Wang et al. reported nine new indolocarbazole alkaloids from the marine bacterium *Streptomyces* sp. DT-A61 [40]. Among these, compound **13** (Figure 7) was shown to inhibit ROCK2 (IC_50_ = 5.7 nM) [40]. The results of biological activity tests showed compound **14** (Figure 7) to be cytotoxic to PC3 human prostate cancer cells (IC_50_ = 0.16 μM) [40]. In 2017, Xiang-Wei Cheng et al. isolated eight known and one new indolocarbazole alkaloid, 12-*N*-methyl-k252c (**15**, Figure 7), from the marine bacterium *Streptomyces* sp. A22 [41]. From this sample set, only **15** reportedly inhibited protein kinases (IC_50_ = 0.91–1.84 μM against various targets) [41].

In 2018, Biao Zhou et al. 15 staurosporine derivatives from the marine bacterium *Streptomyces* sp. NB-A13 [42]. Among these, six were new (**16**–**21**; Figure 8), and each one except for **16** inhibited PKC-θ (IC_50_ 0.06 to 9.43 μM) [42]. The most active of them was compound **21**, which also inhibited SW-620 cells in vitro (IC_50_ = 9.99 nM) at even greater potency than staurosporine (IC_50_ = 25.10 nM) [42]. Structurally, compound **21** bears an additional hydroxy group in the bis-indole ring system that is shared with staurosporine, indicating one possible avenue for medicinal chemistry optimization of other molecules in this chemical class.

From the marine bacterium *Streptomyces* sp. XMA39, Yong-Jun Jiang et al. in 2018 isolated the known compound medermycin, and four new structurally related naphthoquinones, strepoxepinmycins A–D [43]. All of these marine natural products were growth inhibitors for different human pathogenic bacteria, and compound **22** (Figure 9) showed ROCK2 inhibition (IC_50_ > 20 μM) and cytotoxic activity against HCT-116 and PC3 cancer cells in vitro with IC_50_ > 40 mM [43]. 

#### 2.1.2. Kinase Inhibitors from Marine Fungi

**Figure 10:**
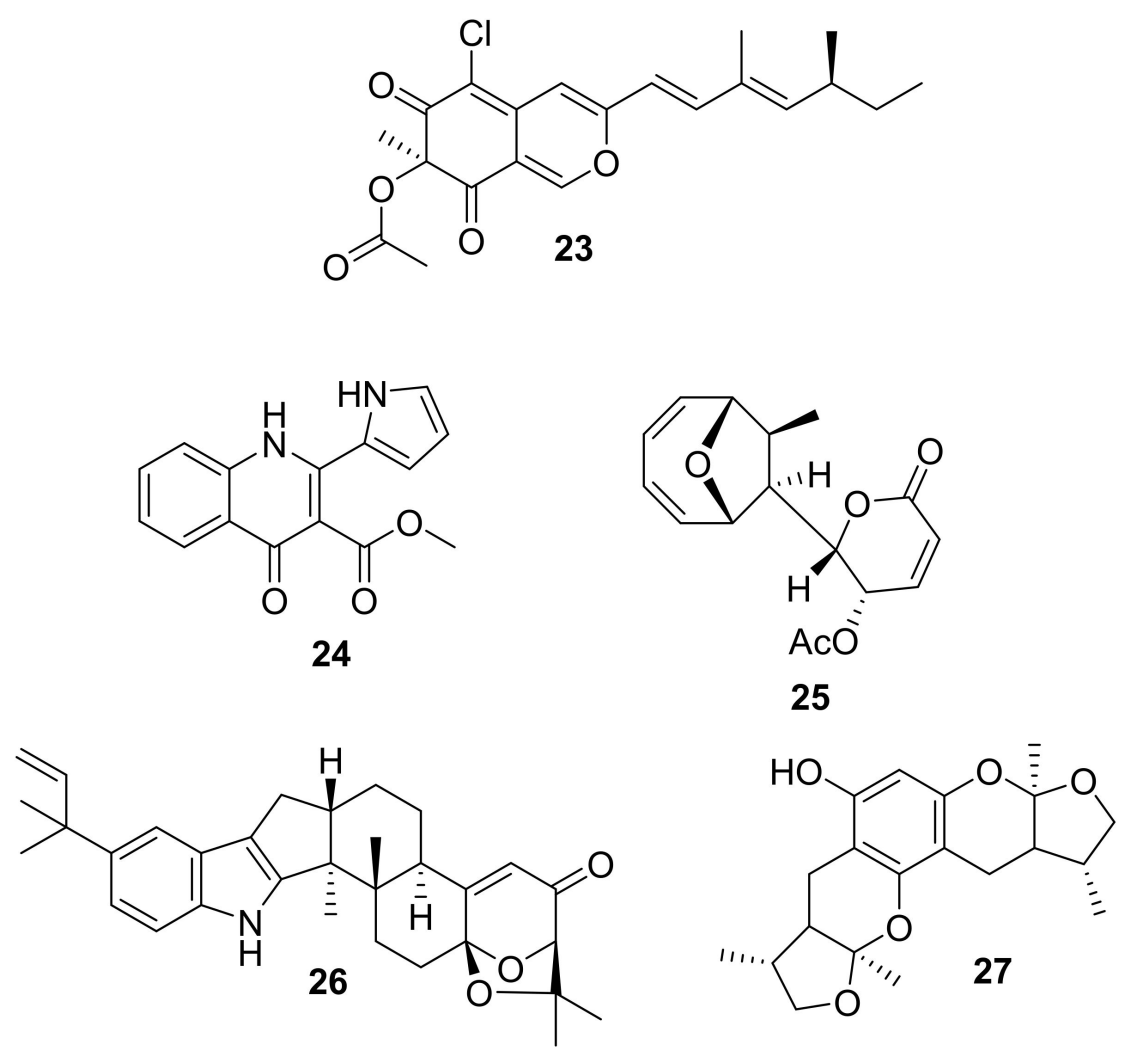
Structure of compounds **23**–**27**.

**Figure 11:**
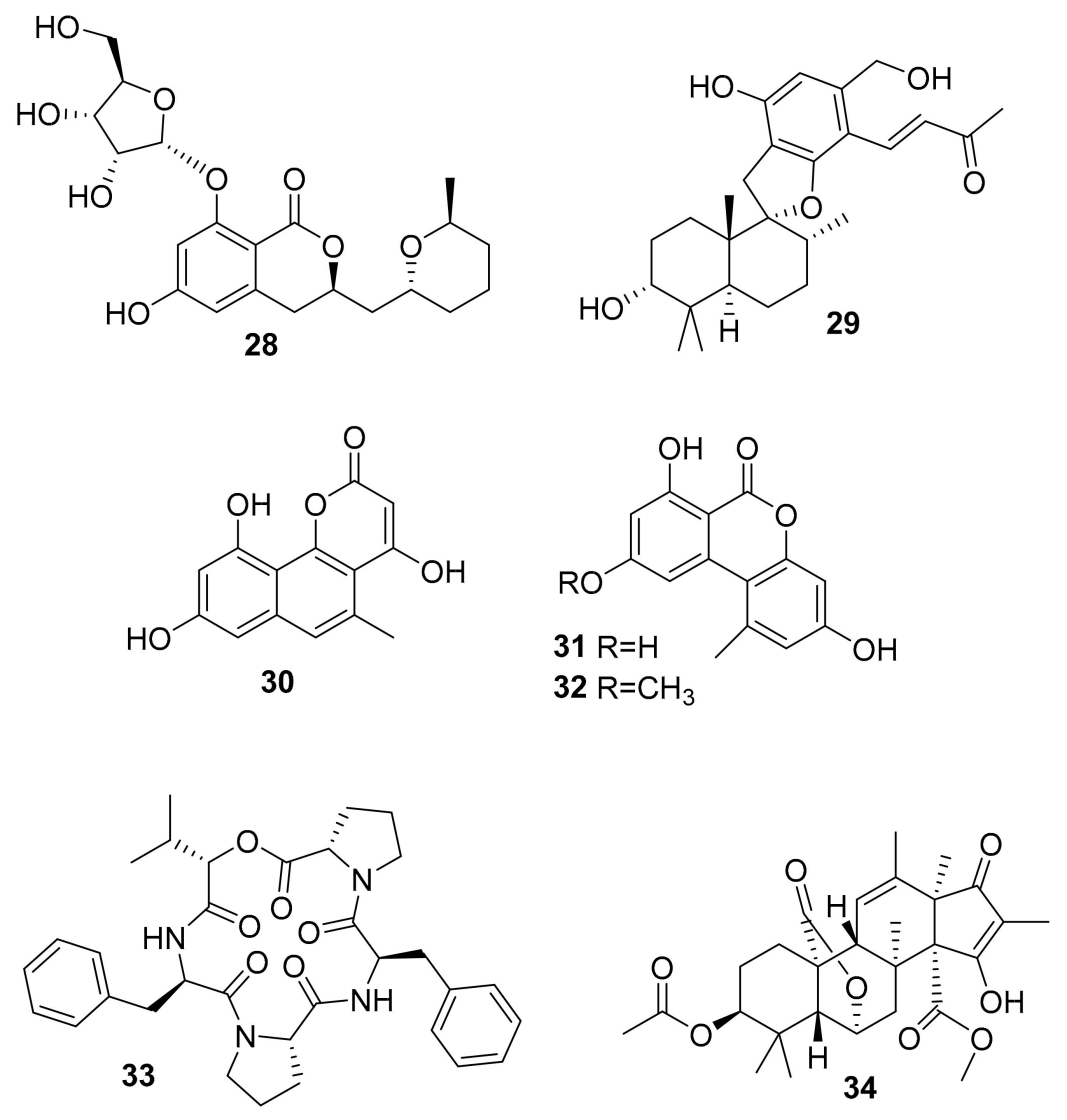
Structure of compounds **28**–**34**.

**Figure 12:**
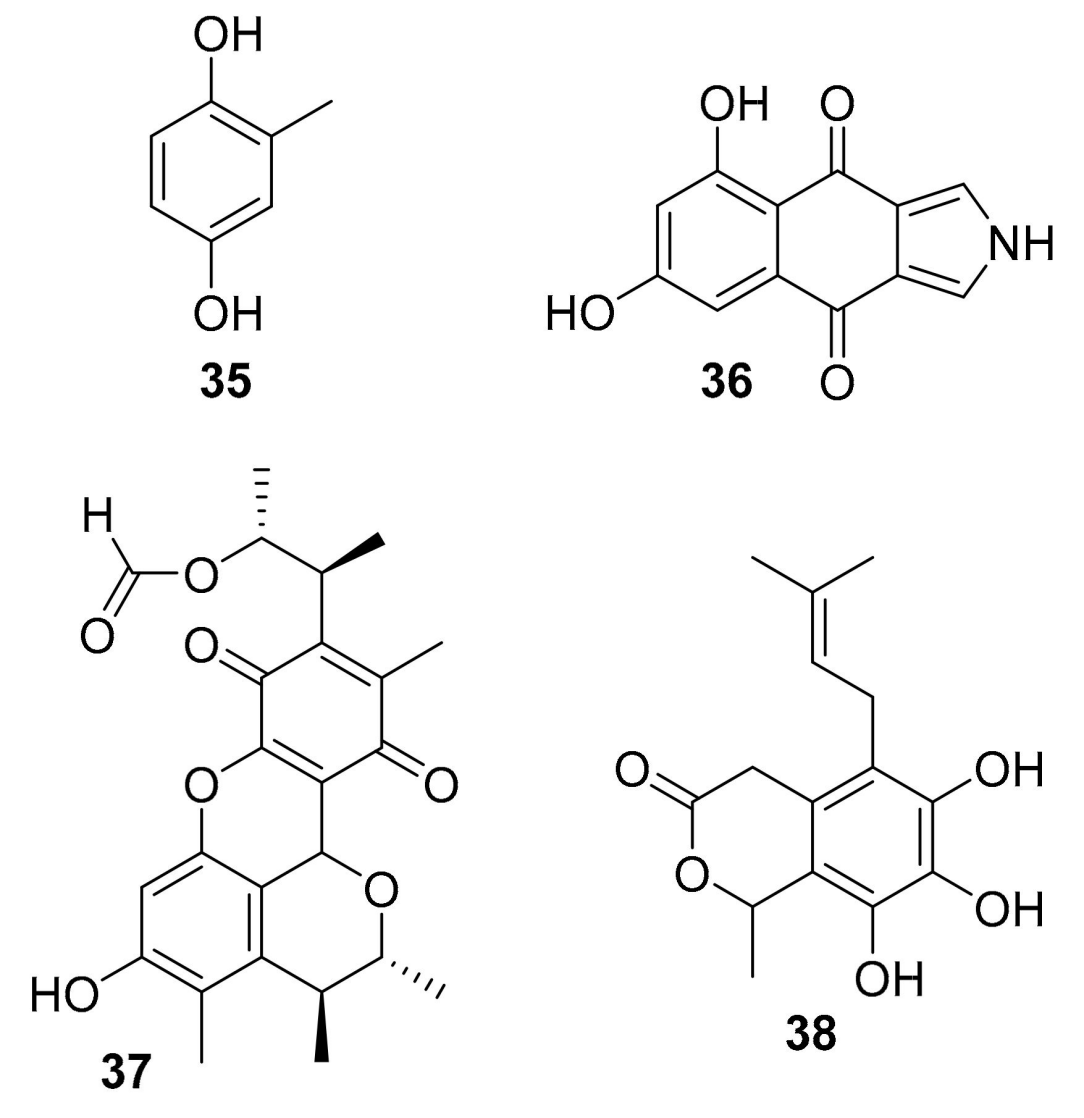
Structure of compounds **35**–**38**.

In the time period covered in this review, 16 new natural product kinase inhibitors (**23–38**) were reported after being isolated from marine fungi. These are listed in Table 2 and shown by structure in Figure 10, Figure 11 and Figure 12. The specific kinases reportedly involved in the biological activities of these compounds are described in the following paragraphs. 

In 2017, Dong-Ni Chen et al. reported the structure of sclerotiorin (**23**, Figure 10) isolated from the marine fungus *Penicillium* sp. Strain ZJ27 [44]. At the same time, sclerotiorin was shown to very weakly inhibit PknG (IC_50_ = 76.5 μM) [44]. In 2014, Dong-Cheol Kim et al. reported that methylpenicinoline (compound **24**, Figure 10) was isolated from marine fungus *Penicillium* sp. and inhibited p-p38 MAPK pathways, although no specific activity value was noted [45]. The authors proposed that **24** might be useful for further development toward the treatment of inflammatory and neuroinflammatory diseases, so the results of ongoing work may demonstrate their follow-up. Mycoepoxydiene (**25**, Figure 10) was reported in 2014 by Wen-Jiao Li et al., after it was isolated from the marine fungal strain *Diaporthe* sp. HLY-1 [46]. Compound **25** was shown to induce apoptosis and inhibit phosphorylation of IKK, and it was suggested that the inhibition of IKK activity and human cholangiocarcinoma (CCA) cells such as SK-ChA-1 (IC_50_ =12.5 ± 0.2 μM) and Mz-ChA-1 (IC_50_ = 35.6 ± 0.2 μM) may fully or partially explain the observed apoptosis [46]. These results suggest that compound **25** could be used as a lead molecule for the design and development of potent and selective new inhibitors of IKK with a potential for therapy of human cholangiocarcinoma disease [15,46]. In 2014, Kunlai Sun et al. reported a new indole-diterpenoid (**26**, Figure 10) that was isolated from the marine fungus *Aspergillus flavus* OUCMDZ-2205 [47]. This compound was found to inhibit PKC-β (IC_50_ = 15.6 μM), at 10 μM arrested A549 cells in the cell cycle S phase, and showed antibacterial activity against *Staphylococcus aureus* (MIC = 20.5 μM) [47]. In 2015, Wen-Liang Chen et al. isolated xyloketal B (compound **27**, Figure 10) from the fungus *Xylaria* sp. (No. 2508) that was collected in a mangrove [48]. Compound **27** was shown at 300 μM to inhibit p-Akt (inhibition rate ~ 34%) and p-ERK1/2 (inhibition rate ~ 40%) protein expression in vitro, and the determinants of anti-proliferation and migration effects against glioblastoma U251 cells (IC_50_ = 287.1 ± 1.0 μM) in vitro included TRPM7-regulated PI3K/Akt and MEK/ERK signaling [48]. 

In 2015, Dong-Cheol Kim et al. [49] reported a new dihydroisocoumarin derivative (compound **28**, Figure 11) from the marine fungus *Aspergillus* sp. SF-5976, and this molecule inhibits p-p38 MAPK [49]. In 2016, Jong Won Kim et al. reported the discovery of stachybotrysin (**29**, Figure 11), which was isolated from the marine fungus *Stachybotrys* sp. KCB13F013 and found to inhibit the RANKL-induced activation of p-ERK, p-JNK and p-p38 MAPK [50]. The kinase inhibition activities of compound **28** and **29** were determined by Western blot analyses. In 2016, Jutta Wiese et al. reported pannorin (**30**), alternariol (**31**), and alternariol-9-methylether (**32**), which were isolated from the marine fungus, *Botryotinia fuckeliana* [51]. Compounds **30**–**32** (Figure 11) showed potent inhibition of GSK-3 in vitro with IC_50_ = 0.35 ± 0.04 μM, 0.13 ± 0.04 and 0.20 ± 0.04 μM, respectively [51]. The highly oxygenated benzocoumarin scaffolds represented in these molecules are apparently effective groups for GSK-3β inhibition, and these could merit further development through medicinal chemistry approaches although the current leads show very weak antibacterial and cytotoxic effects. In 2015, Wonmin Ko et al. reported alternaramide (**33**, Figure 11), which was isolated from the marine fungus *Alternaria* sp. SF-5016 [52]. This compound inhibited the formation of p-JNK and p-p38 MAPK, as determined by Western blotting, suggesting that it could be useful in the treatment of various acute, systemic, and neurological inflammatory diseases [52]. In 2015, Kwang-Ho Cho et al. reported citreohybridonol (**34**, Figure 11) after it had been isolated from the extract of the marine-derived fungus *Toxicocladosporium* sp. SF-5699 [53]. Compound **34** was pursuantly shown to inhibit the activation of p38 MAPK pathways by the Western blot analysis [53].

In 2016, M García-Caballero et al. reported that toluquinol (**35**, Figure 12), isolated from the marine fungus *Penicillium* sp. HL-85-ALS5-R004, inhibited the phosphorylation of p-Akt and p-ERK1/2 in vitro and VEGF-C-induced lymphatic vessel formation and corneal neovascularization in mice [54]. Compound **35** plays a demonstrated role in inhibiting angiogenesis and lymphangiogenesis, which is meaningful in connection with the implications of tumour-induced lymphangiogenesis and lymphatic metastasis. In 2016, Bin Wu et al. reported on biscogniauxone (**36**, 12), a new isopyrrolonaphthoquinone isolated from the deep-sea (2800 m) fungus *Biscogniauxia mediterranea* [55]. Compound **36** moderately inhibited GSK-3β (IC_50_ = 8.04 μM) and had almost negligible antibacterial activity against *Staphylococcus epidermidis* and methicillin-resistant *S. aureus* (IC_50_ ~ 100 μM) [55]. Marine-derived fungi from the deep sea are promising but severely under-studied biological resources, and these possess abundant novel and bioactive secondary metabolites that urgently need to be mined if adequate access to the resource can be obtained [57]. In 2017, Nguyen Thi Thanh Ngan et al. reported that citrinin H1 (compound **37**, Figure 12), isolated from the marine fungal strain *Penicillium* sp. SF-5629, inhibited p-p38 MAPK expression in vitro [56]. Additionally in 2017, Marion Navarri et al. reported that dihydrosecofuscin (compound **38**, Figure 12), isolated from a deep-sea (765 m) fungus *Oidiodendron griseum* UBOCC-A-114129, inhibited CLK1 (IC_50_ = 15.6 μg/mL) [58]. Compound **38** showed weak antibacterial activities (MIC ~ 100 μg/mL) against Gram-positive bacteria, and the authors noted a potential use of this CLK1 inhibitor for the treatment of Alzheimer’s due to the connection between the particular kinase and disease [58].

#### 2.1.3. Kinase Inhibitors from Marine Soft Coral 

**Figure 13:**
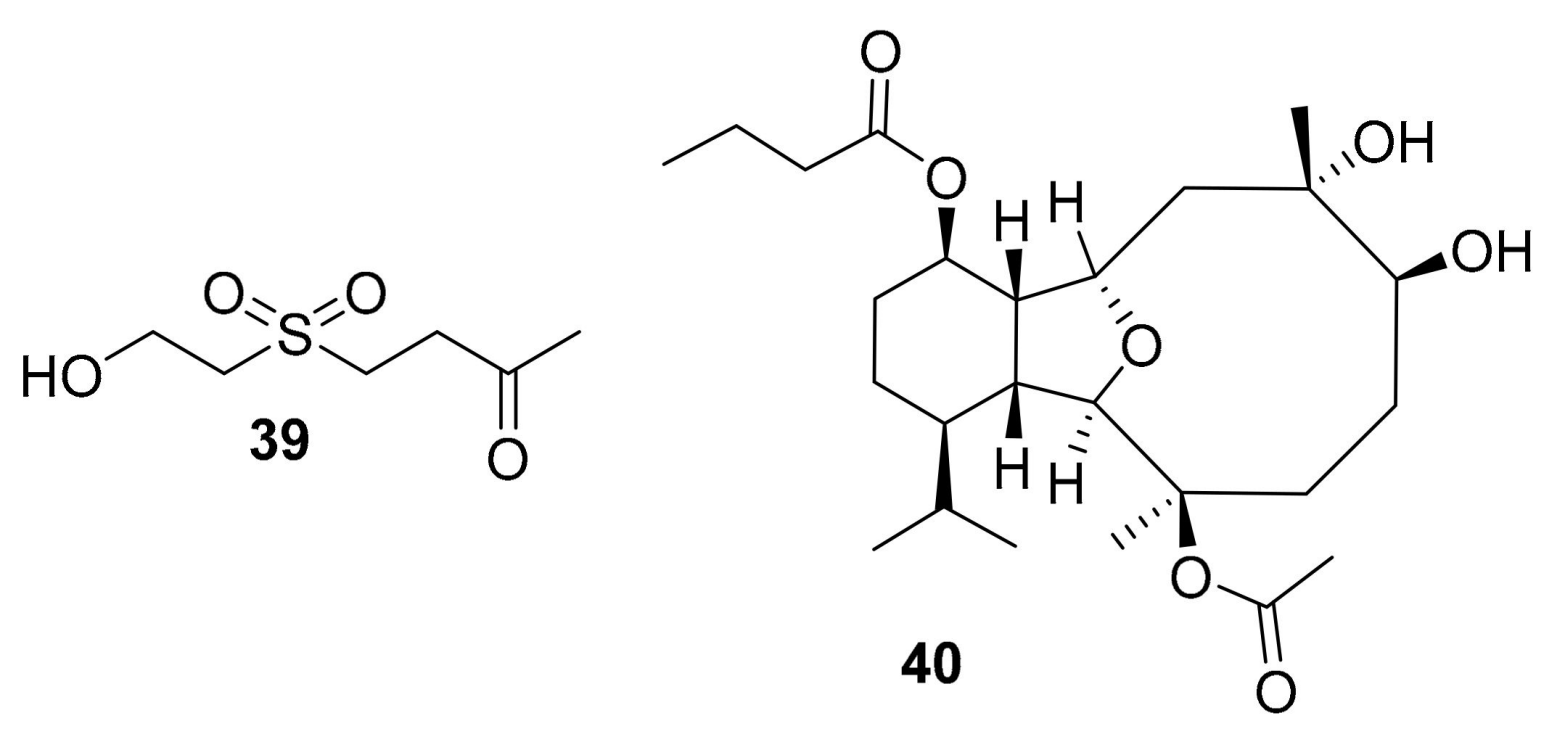
Structure of compounds **39**–**40**.

In the time period covered in this review, two new natural product kinase inhibitors (**39** and **40**) were reported after being isolated from marine soft coral (see Table 3). Dihydroaustrasulfone alcohol (**39**, Figure 13), is a molecule that was reported in 2015 by Pei-Chuan Li et al. after being isolated from soft corals [59]. This compound inhibited ERK/MAPK and PI3K/AKT, and was suggested as a lead that could be pursued for the prevention and treatment of arterial restenosis [59]. In 2017, pachycladin A (**40**, Figure 13) was re-isolated from the soft coral *Cladiella pachyclados* found in the Red Sea, and a newly shown activity (absent from the previous report of this compound in 2010 [61]) was that it inhibited two members of the EGFR family when screened at 10 μM [60]. 

#### 2.1.4. Kinase Inhibitors from Marine Animals 

**Figure 14:**
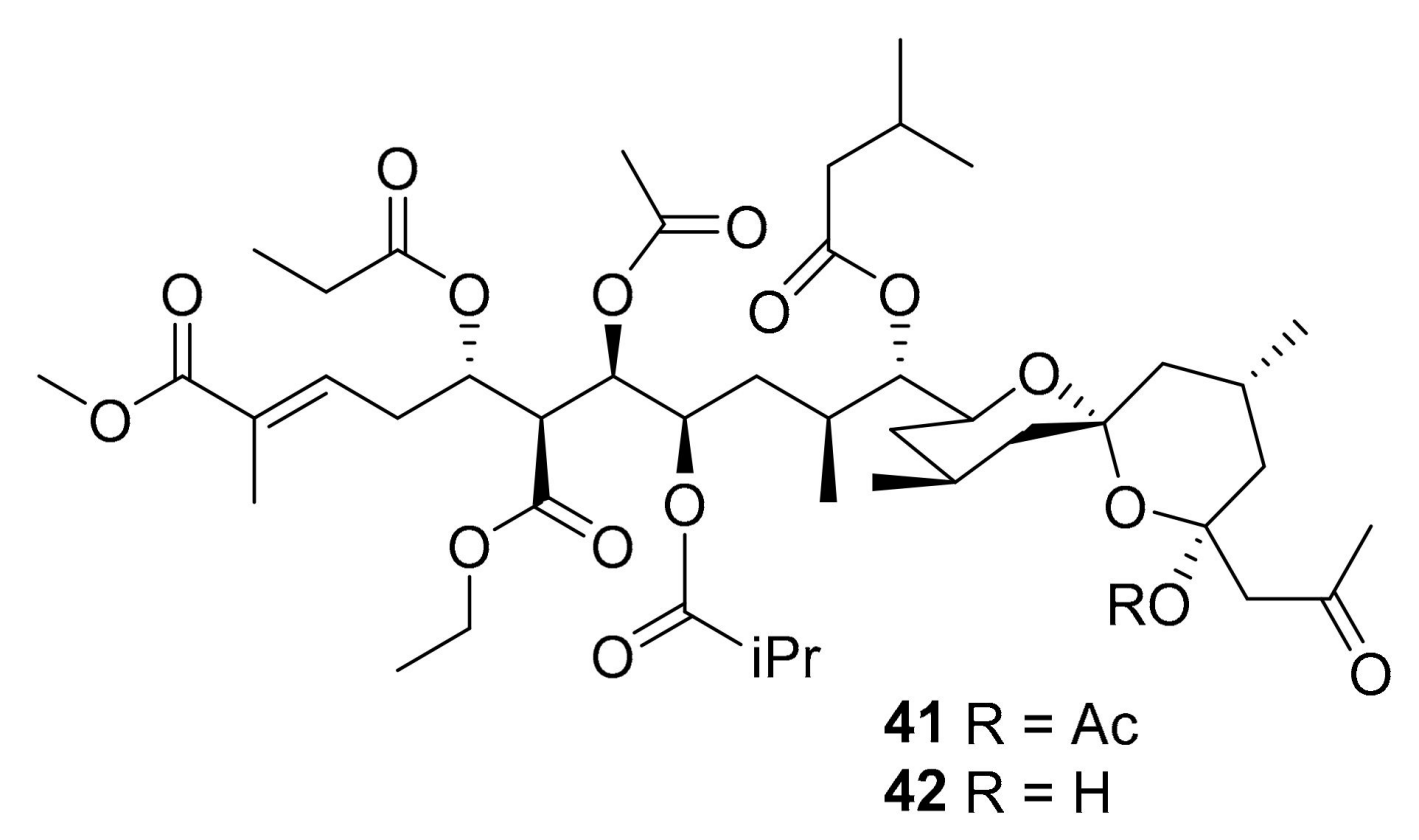
Structures of didemnaketals D (**41**) and E (**42**).

**Figure 15:**
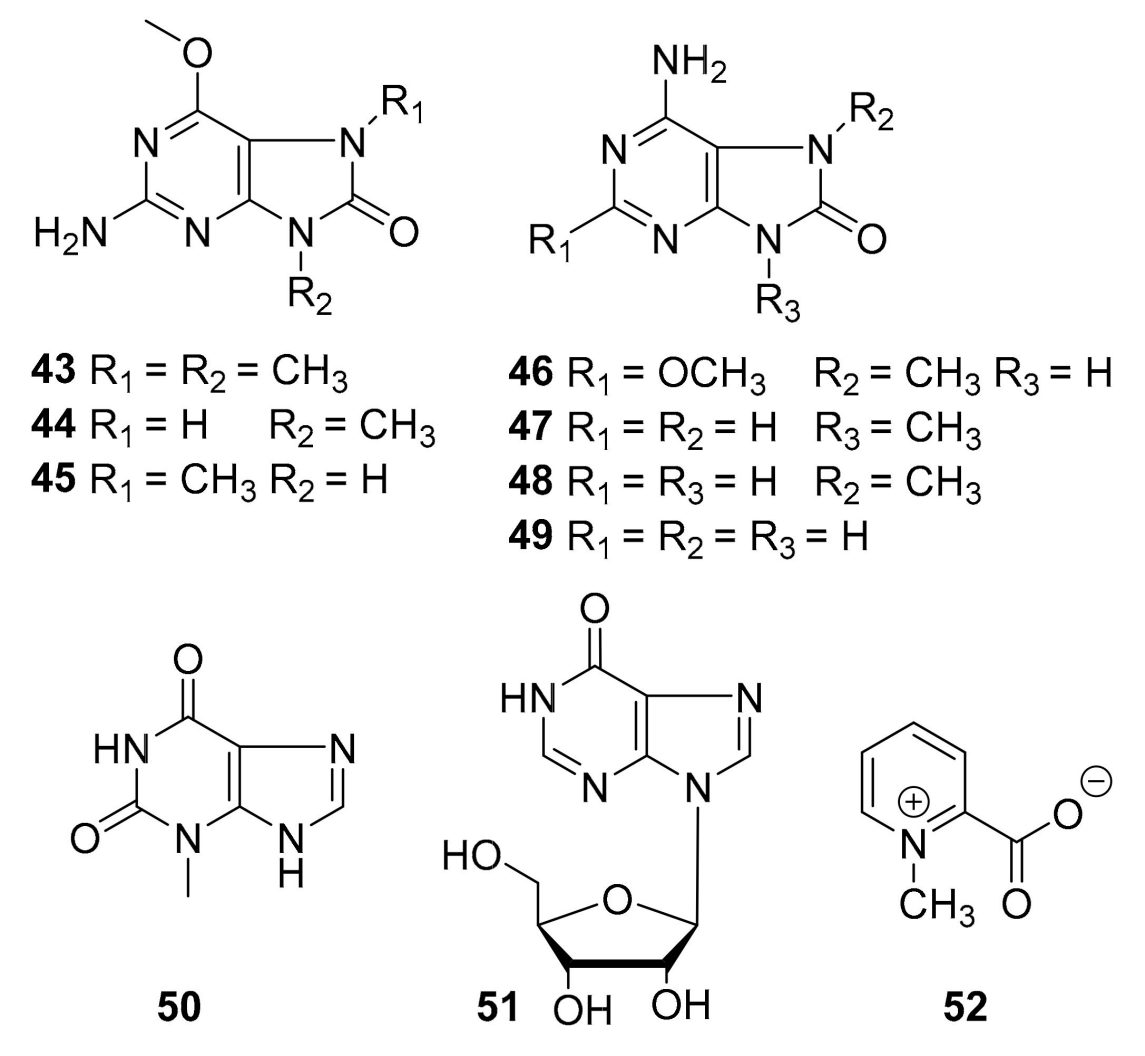
Structures of purine and pyridine alkaloids (**43**–**52**).

**Figure 16:**
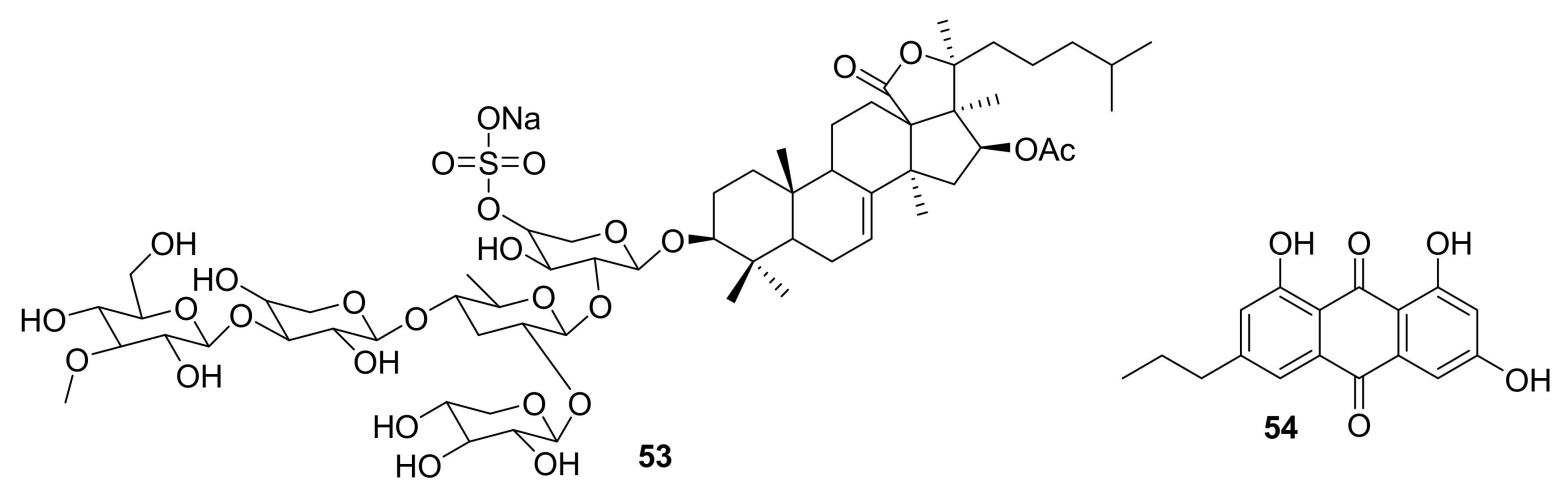
Structure of compounds **53**–**54**.

In the time period covered in this review, 14 new natural product kinase inhibitors (**41–54**) were reported after being isolated from marine animals. These are listed in Table 4 and shown by structure in Figure 14, Figure 15 and Figure 16. The specific kinases reportedly involved in the biological activities of these compounds are described in the following paragraphs. 

In 2014, didemnaketals D and E (compounds **41** and **42**, Figure 14) were reportedly discovered from marine tunicates of the genus *Didemnum*, and these compounds inhibited several kinases (CDK5, CK1, DyrK1A, and GSK3) at 10 μg/mL and were moderately antimicrobial in vitro against *S. aureus* and *Bacillus subtilis* [62]. In 2015, Youssef [63] et al. reported the three new purine alkaloids **43**–**46** (Figure 15), together with seven known compounds (**45**–**52**, Figure 15) from the marine tunicate *Symplegma rubra*, collected from the Saudi Red Sea coast (5–7 m depth). The authors reported that these compounds were found to be moderate inhibitors of different kinases (CDK5, CK1, DyrK1A, and GSK-3), with some activity at 10 μg/mL, but noted that the observed IC_50_ values (>10 μM) were “not sufficient to pursue with these compounds into the in vivo studies” [63]. This report was somewhat vague and did not specify the individual activity of each molecule.

In 2017, frondoside A (compound **53**, Figure 16) was isolated from *Cucumaria frondosa*, the Atlantic sea cucumber, and reported as being an effective inhibitor of PAK1 (IC_50_ ~ 1.2 μM), LIMK (IC_50_ ~ 60 μM), AKT (IC_50_ ~ 59 μM) and A549 lung cancer cells (IC_50_ ~ 1–3 μM) in vitro, indicating that it may be useful in the treatment of malignancies [64,65]. Additionally in 2017, the molecule 1-deoxyrhodoptilometrin (compound **54**, Figure 16), which was first discovered by Wright et al. from the Echinoderm *Colobometra perspinosa* [67], was re-isolated from the marine echinoderm *Comanthus* sp. [66]. This compound was subsequently reported to inhibit the IGF1-R kinase, focal adhesion kinase, and EGF receptor kinase with IC_50_ = 5, 8.4 and 4 μM, respectively [66].

#### 2.1.5. Kinase Inhibitors from Marine Algae

**Figure 17:**
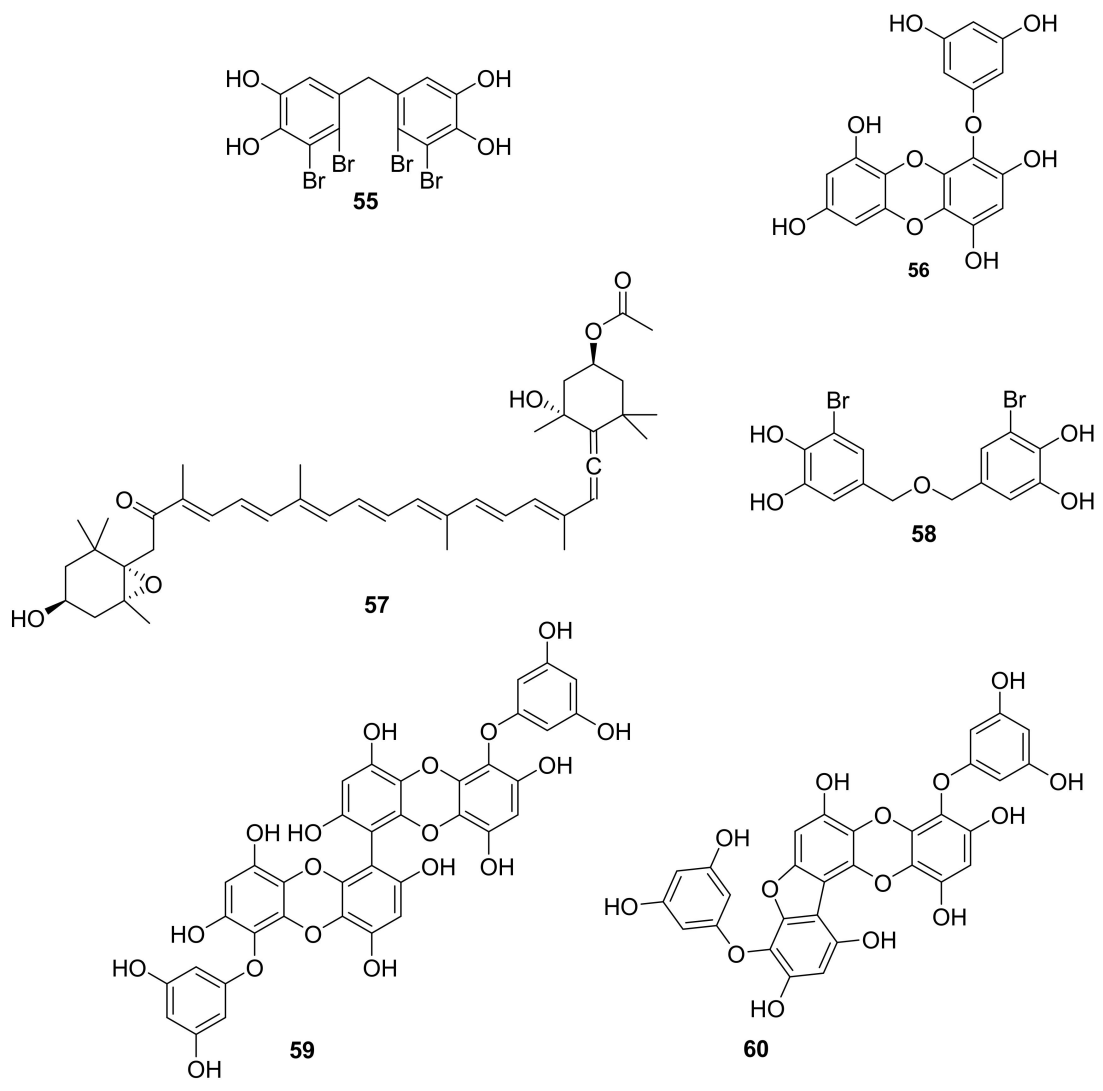
Structure of compounds **55**–**60**.

**Scheme 1:**
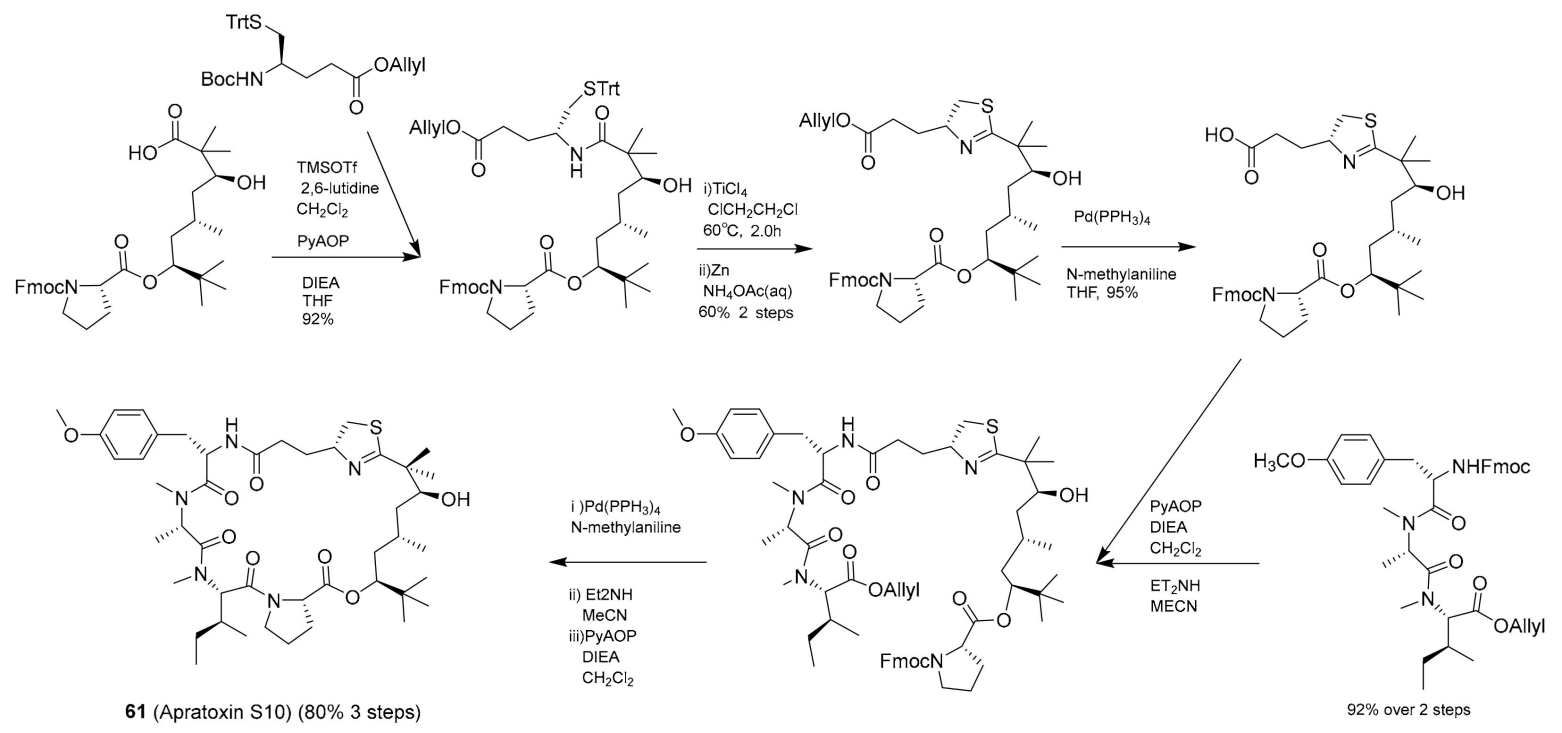
Synthesis and structure of apratoxin S10 (**61**).

**Figure 18:**
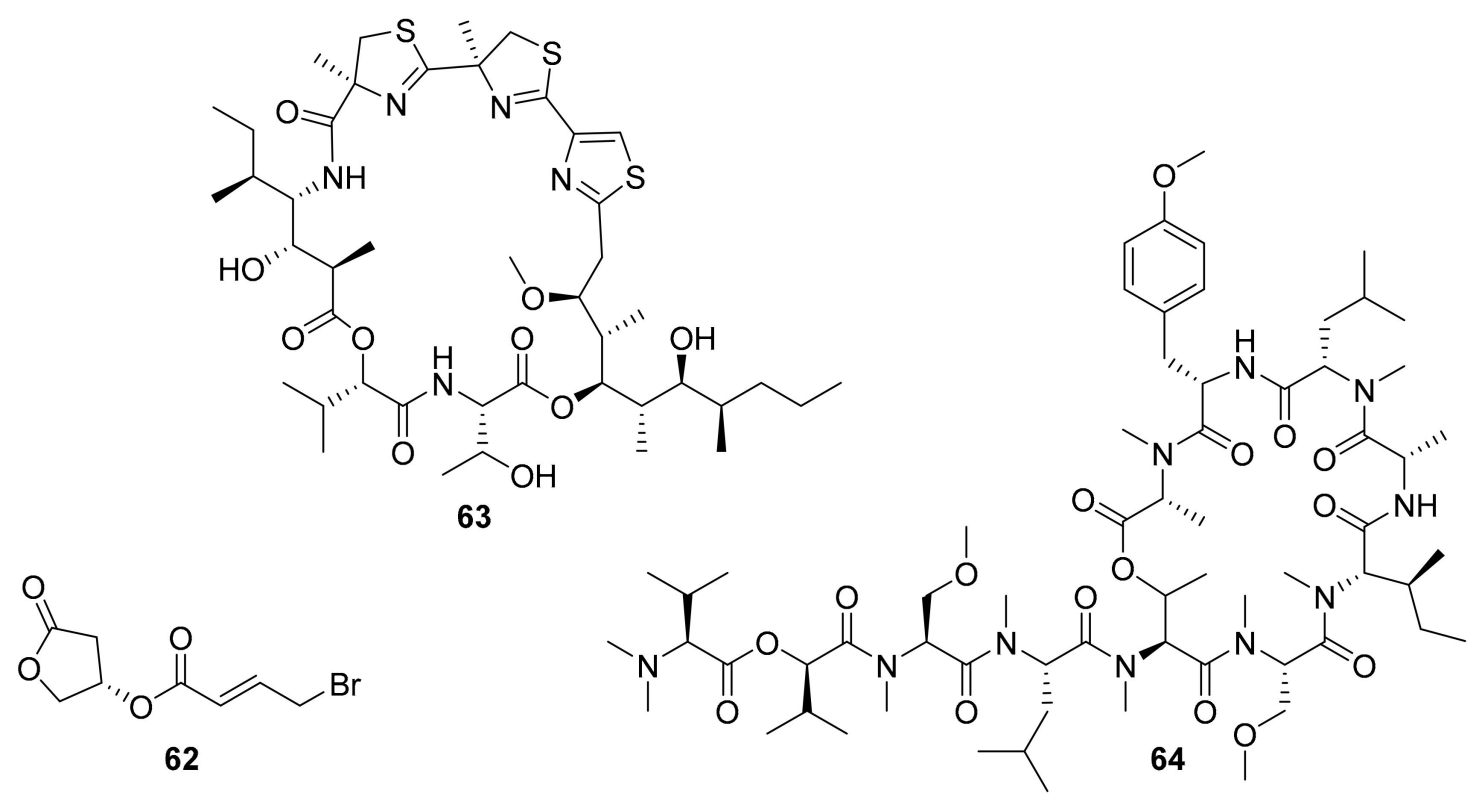
Structure of compounds **62**–**64**.

In the time period covered in this review, 10 new natural product kinase inhibitors (**55–64**) were reported after being isolated from marine algae. These are listed in Table 5 and shown by structure in Figure 17, Figure 18 and Scheme 1. The specific kinases reportedly involved in the biological activities of these compounds are described in the following paragraphs. 

In 2015, Shuai-Yu Wang et al. reported compound **55** (Figure 17) as a multi-target RTKs inhibitor such as FGFR2, FGFR3, VEGFR2 and PDGFR-α (with in vitro inhibition rates of 57.7%, 78.6%, 78.5% and 71.1%, respectively), which was isolated from *Rhodomela confervoides*, a red alga, and this molecule also inhibits p-PKB/Akt [68,69]. Compound **55** may accordingly present a scaffold from which to develop new multi-target RTKs inhibitors. In 2017, Sung-Hwan Eom et al. reported that the polyphenolic molecule eckol (**56**, Figure 17), purified from the edible brown seaweed *Eisenia bicyclis*, inhibited *Propionibacterium acnes* mediated phosphorylation of Akt [70]. Eckol is also proposed as an anti-inflammatory agent and antioxidant. In 2017, fucoxanthin (**57**, Figure 17), a well-known marine carotenoid, was shown to inhibit Akt/NF-κB and involved MAPK pathways in vitro, suggesting that it could be a potential therapeutic agent acting by this mechanism for the therapy of neurodegenerative diseases [71,72,73]. Further validation studies are obviously necessary, and likely ongoing. In 2018, Youn Kyung Choi et al. reported the isolation of bis (3-bromo-4,5-dihydroxybenzyl) ether (**58**, Figure 17) from *Polysiphonia morrowii*, a red alga [74]. Compound **58** selectively inhibited p-ERK, demonstrating that it could have potential in the treatment of inflammatory diseases [74]. In 2014, Paudel et al. postulated that since the polyphenolics 6–6 bieckol and pholorofucofuroeckol A (**59** and **60**, Figure 17) inhibited p-ERK1/2 and p-JNK activation, these hold potential value for treating pulpitis and oral diseases [75]. These two compounds were isolated from *Eisenia bicyclis*, a brown alga [75].

Apratoxin A is a cyanobacterial, or “blue-green algal”, natural product macrocycle that was first reported by Luesch et al. in 2001 as a product of a hybrid polyketide synthase-nonribosomal peptide synthetase (PKS/NRPS) biosynthetic pathway [83]. In 2017, Wei-Jing Cai et al. later designed and synthesized a series of cyclic depsipeptides based on apratoxin A, including apratoxin S10 (**61**, Scheme 1) [76]. This analogue of apratoxin A was selected as a desirable drug lead with cancer cell growth (IC_50_ = 5.97 nM against HCT116) and RTKs inhibitory activity [76]. The synthetic route of generating apratoxin S10 is depicted in Scheme 1. The synthesis of apratoxin S10 followed the reported strategy used to produce the lead compound, apratoxin A, with some modification to introduce new functionality [76].

In 2015, Mahesh Sapkota [78] et al. reported bromo-honaucin A (**62**, Figure 18), which was first isolated from marine cyanobacterium *Leptolyngbya crossbyana* and chemical synthesis was completed by Hyukjae Choi and William H. Gerwick et al. [77]. Compound **62** inhibited AKt and ERK, and the authors noted a potential use of this AKt and ERK inhibitor for the treatment of bone lysis due to the connection between the particular kinase and disease. In 2015, Zhengyu Cao et al. [81] reported the discovery of hoiamide A (**63**, Figure 18), which was first isolated from marine cyanobacterium *Moorea producens* by Alban Pereira et al. [79] and found to stimulate and increase the hoiamide A-induced activation of JNK. Compound **63** showed the inhibition of neurite outgrowth (IC_50_ = 4.89 nM). In 2016, Jeffrey D. Serrill et al. [82] reported coibamide A (**64**, Figure 18) (first isolated from marine cyanobacterium *Leptolyngbya* sp. [81]) inhibited VEGFA/VEGFR2 expression in mice and showed antitumor activities against glioblastoma xenografts. 

#### 2.1.6. Kinase Inhibitors from Marine Sponges 

In the time period covered in this review, 42 new natural product kinase inhibitors (**65–106**) were reported after being isolated from marine sponges. These are listed in Table 6, shown by structure in Figure 19, Figure 20, Figure 21, Figure 22, Figure 23, Figure 24, Figure 25 and Figure 26 and Scheme 2, Scheme 3, Scheme 4 and Scheme 5, and described in detail in the following paragraphs. 

**Figure 19:**
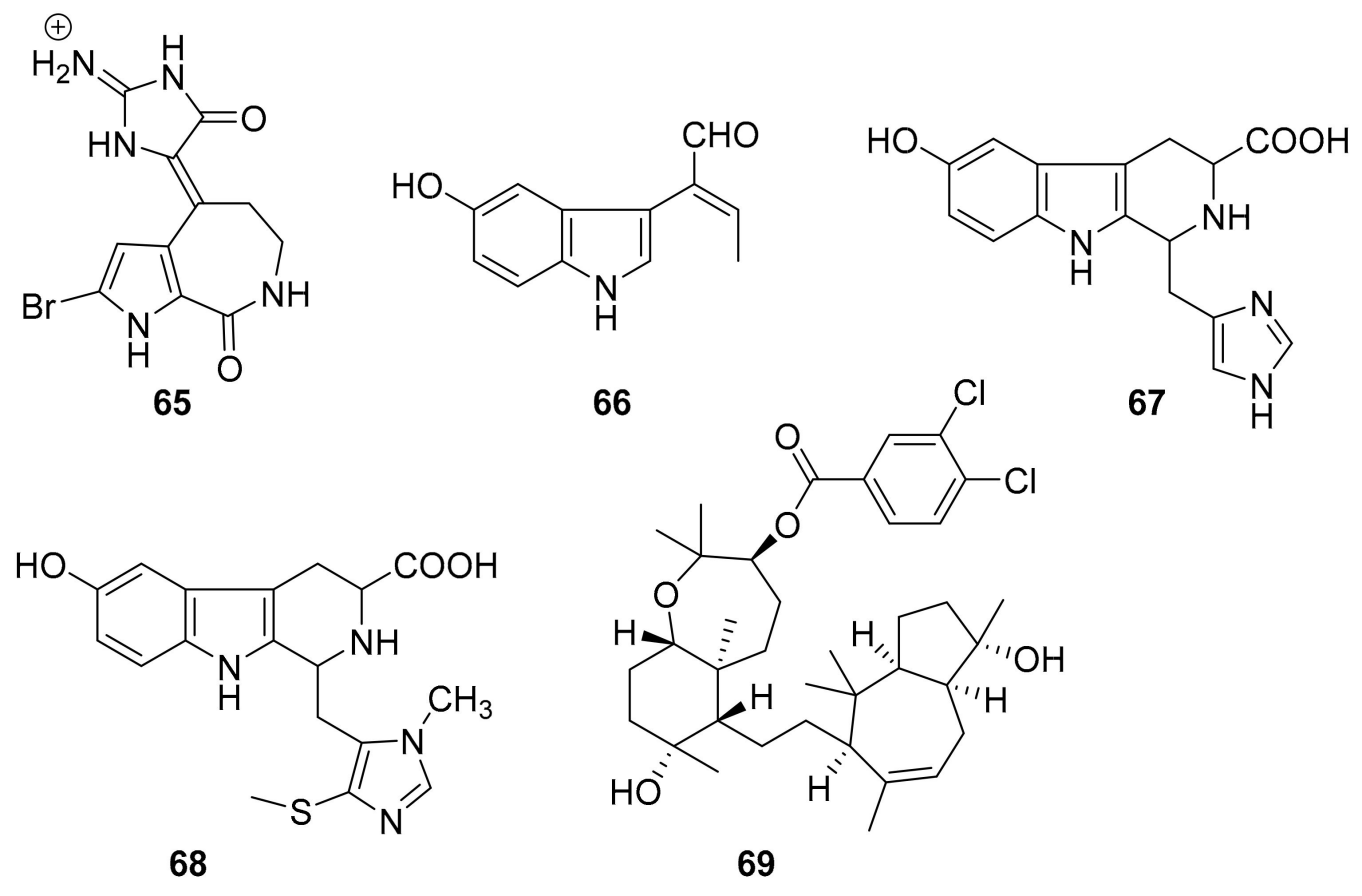
Structure of compounds **65**–**69**.

In 2014, Fabien Plisson et al. reported some new bromopyrrole alkaloids including hymenialdisine (**65**, Figure 19) that were isolated from *Callyspongia* sp. [84]. This interesting class of molecules has been studied for several decades, owing to their peculiar structural skeleton and associated pharmacological effects [85,86]. Compound **65** inhibited CK1, CDK5, GSK3β and SW620 (3.1 μM), KB-3-1 (2.0 μM), which are targets for treating neurodegenerative diseases [84]. In 2014, the β-carboline alkaloids **66**–**68** (Figure 19), including hainanerectamines B and C, were isolated from the marine sponge *Hyrtios erecta* after it was collected in Hainan, China [87]. These molecules have been shown to inhibit the dual kinase Aurora A with relatively high IC_50_ values of 10 μg/mL–25 μg/mL [88]. In 2014, Mohamed R. Akl et al. reported the isolation of sipholenol A from the sponge *Callyspongia siphonella*, collected in the Red Sea [89]. A semisynthetic analogue of sipholenol A, sipholenol A-4-*O*-3′,4′-dichlorobenzoate (**69**, Figure 19) was associated with the suppression of the Brk and FAK signaling pathway in vitro and in vivo, also inhibited MDA-MB-231, MCF-7, BT-474 and T-47D breast cancer cells with IC_50_ values of 7.5 μM, 15.2 μM, 20.1 μM and 25.1 μM, respectively, making it a potentially interesting pro-drug for sipholenol A [89].

**Figure 20:**
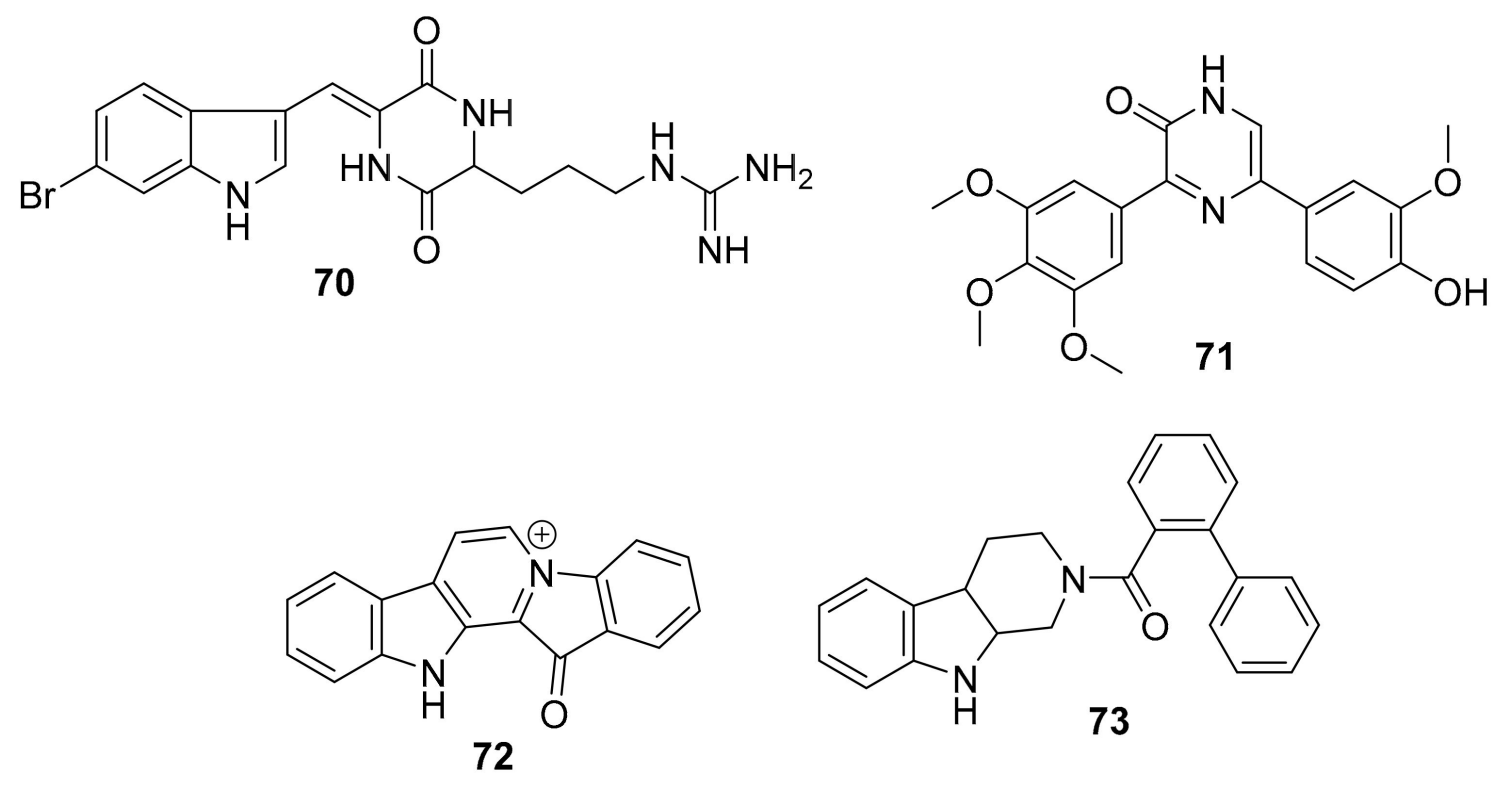
Structure of compounds **70**–**73**.

**Scheme 2:**
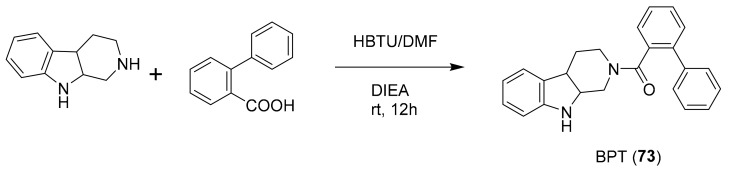
Synthetic preparation of the fascaplysin derivative, compound **73**.

**Scheme 3:**
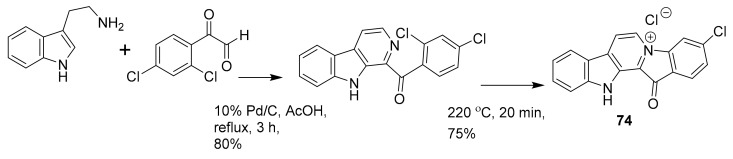
Synthesis of 4-chloro fascaplysin (**74**).

In 2015, Karianne F. Lind et al. reported that barettin (**70**, Figure 20), which was first isolated and described in 1986 [90], inhibited RIPK2 (IC_50_ = 8.0 μM), CAMK1a (IC_50_ = 5.7 μM), SIK2 (IC_50_ = 6.1 μM) and produced anti-inflammatory effects in vitro [91,92]. In 2015, Rebecca Horbert et al. reported the synthetic compound **71** (Figure 20) with a 3,5-diarylpyrazin-2-one core that was based on hamacanthins A and B, which are sponge natural products of the bis-indole alkaloid class [93]. Compound **71** inhibited PDGF-Rβ with good potency (IC_50_ = 0.02 μM), and was cytotoxic in vitro against PDGF-R dependent cancer cells [93]. The accompanying structure-activity relationship indicated that the added 3′-methoxy and 4′-hydroxyphenyl substituents led to an increase in the inhibitory activity against PDGF-Rβ, helping to direct future related research studies [93]. Fascaplysin (**72**, Figure 20) previously was isolated from the marine sponge *Fascaplysinopsis* species [94] and shown to inhibit CDK-4/D1 with moderate potency (IC_50_ = 0.35 μM) and some selectivity amongst kinases [95]. This compound is known to have multiple other mechanisms of action, including DNA binding. More recently, in 2015, S. Mahale et al. synthesized a non-planar analogue of fascaplysin, *N*-(biphenyl-2-yl) tryptoline (**73**, Figure 20), and reported that this retained some weak but selective inhibition of CDK-4/D1 (IC_50_ ~10 μM) [96]. Although this represents a marked drop in potency, compound **73** was successfully designed to overcome DNA intercalation that had been considered a liability of the more active **72** [96]. Compound **73** was validated as being a preferable anticancer agent than **72**, including the results from PK studies in mice [96]. It has been further suggested that compound **73** constitutes a lead scaffold from which drug candidates could be designed for the treatment of certain cancers [96,97,98]. The synthetic route of production for **73** is depicted in Scheme 2 [96], which includes commercially available starting materials (biphenyl 2-carboxylic acid and tetrahydro-β-carboline), commonly used reagents [hexafluorophosphate benzotriazole tetramethyl uronium (HBTU) and *N*,*N*-diisopropylethylamine (DIEA)], and runs in good yield (~70%). The further development of fascaplysin analogues and derivatives through medicinal chemistry will accordingly help overcome the common “supply problem” of advancement of natural product drug candidates. In 2017, Sonia Sharma et al. synthesized 4-chlorofascaplysin (**74**, Scheme 3; a marine sponge alkaloid fascaplysin analogue) and found it to be cytotoxic to breast cancer cells in vitro (IC_50_ = 0.3 μM) and to inhibit PI3K/AKt/mTOR in vitro and in vivo [99]. The synthetic route of 4-chloro fascaplysin is depicted in Scheme 3, where the two-step synthesis includes low cost materials and high yield reactions [99].

**Figure 21:**
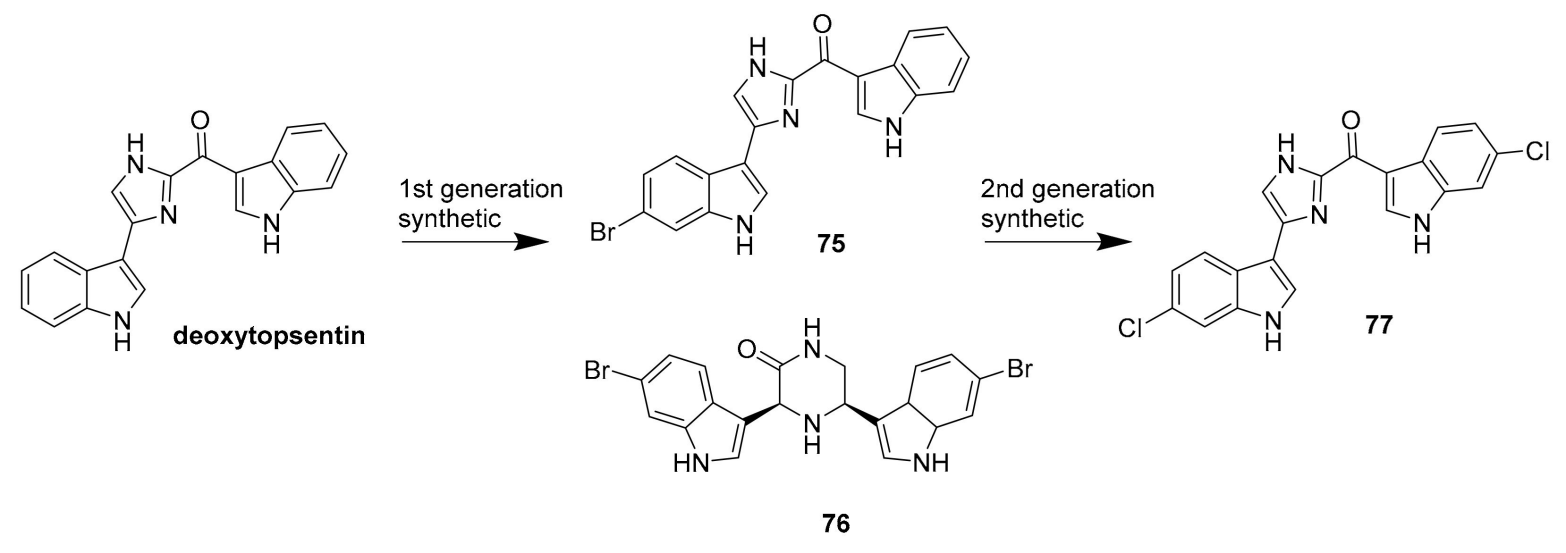
Synthetic analogues (**75**–**77**) of the marine bisindole alkaloid deoxytopsentin.

**Figure 22:**
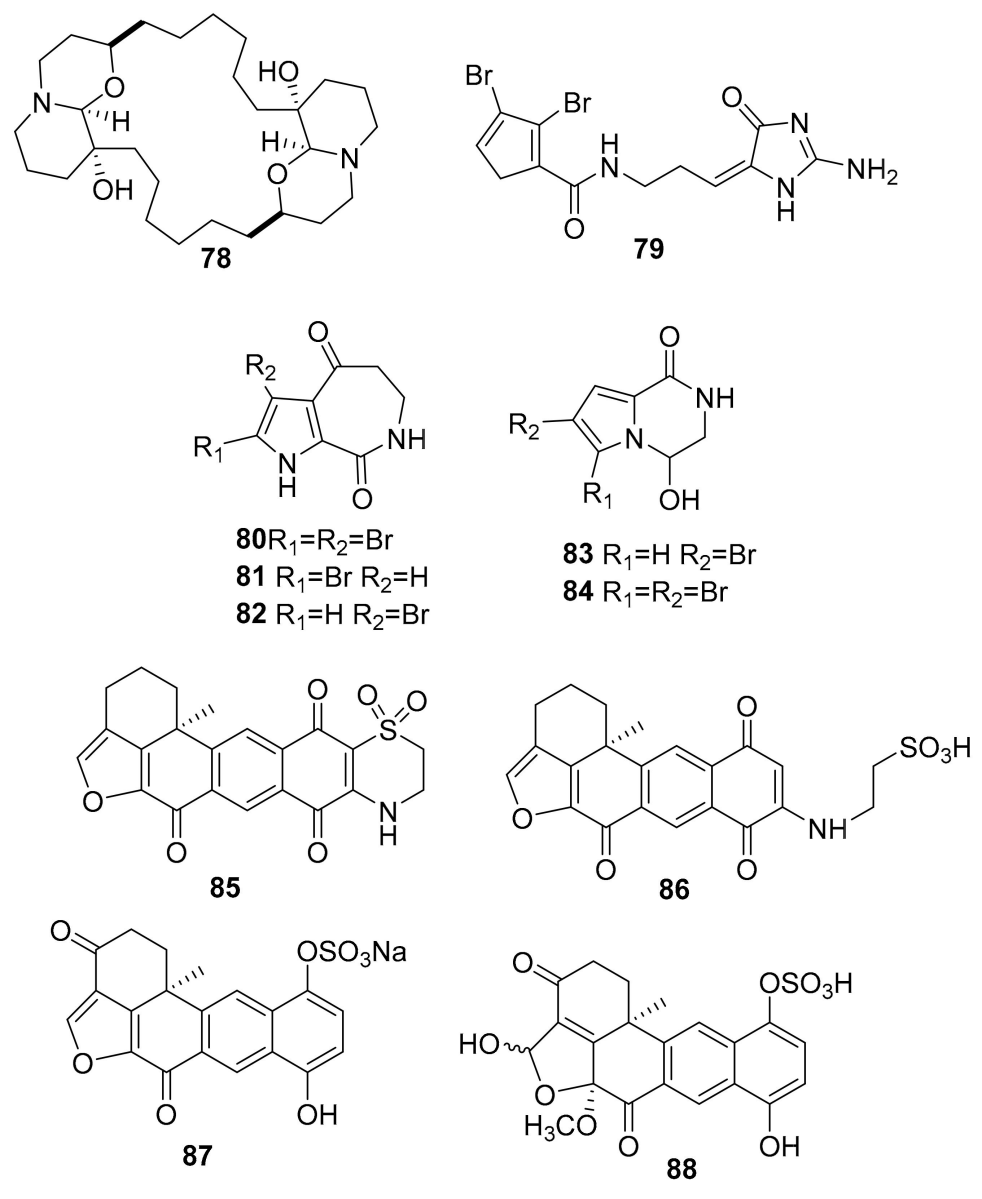
Structure of compounds **78**–**88**.

In 2015, Clinton G. L. Veale et al. created a series of synthetic bisindole compounds including **75** and **76** (Figure 21), based on deoxytopsentin that was isolated from marine sponge *Topsentia pachastrelloides* [100]. Compounds **75** and **76** inhibit PK from methicillin resistant *S. aureus* (IC_50_ = 60 and 16 nM, respectively), and had good in vitro antibacterial activity (MIC ~ 10 μg/mL) [100]. Compound **77** (Figure 21) is among the series of synthetic analogues based on **75** and **76**, and it had enhanced inhibitory activity against MRSA PK (IC_50_ = 1.4 nM) [100]. Additionally in 2015, Mohamed R. Akl et al. reported an RTK inhibitor, araguspongine C (compound **78**, Figure 22), was isolated from the marine sponge *Xestospongia* sp. [101]. Sherif S. Ebada et al. reported the productive discovery of 25 bromopyrrole alkaloids in 2015, from the Indonesian marine sponges *Stylissa massa* and *Stylissa flabelliformis* [102]. This sample set included dispacamide E (compound **79**) [103], the brominated aldisines **80**–**82** [103,104], (–)-mukanadin C (**83**) [105,106] and (–)-longamide (**84**) [107], these compounds, shown in Figure 22, were moderate inhibitors of GSK-3, DYRK1A and CK-1 (IC_50_ 0.6 ~ 6.4 μM), and may have future development potential for treating various diseases [102]. In 2015, Fei He et al. reported seven new derivatives of adociaquinone that were isolated from a marine sponge, *Xestospongia* sp., collected in Indonesia [108]. Among these, compounds **85** and **86** (Figure 22) moderately but selectively inhibited CDK9/cyclin T and CDK5/p25 at 3–6 μM IC_50_, while **87** and **88** (also Figure 22) moderately inhibited most protein kinases without selectivity (IC_50_ = 0.5 ~ 7.5 μM) and compound **88** showed marginal activity against DYRK1A [108].

**Figure 23:**
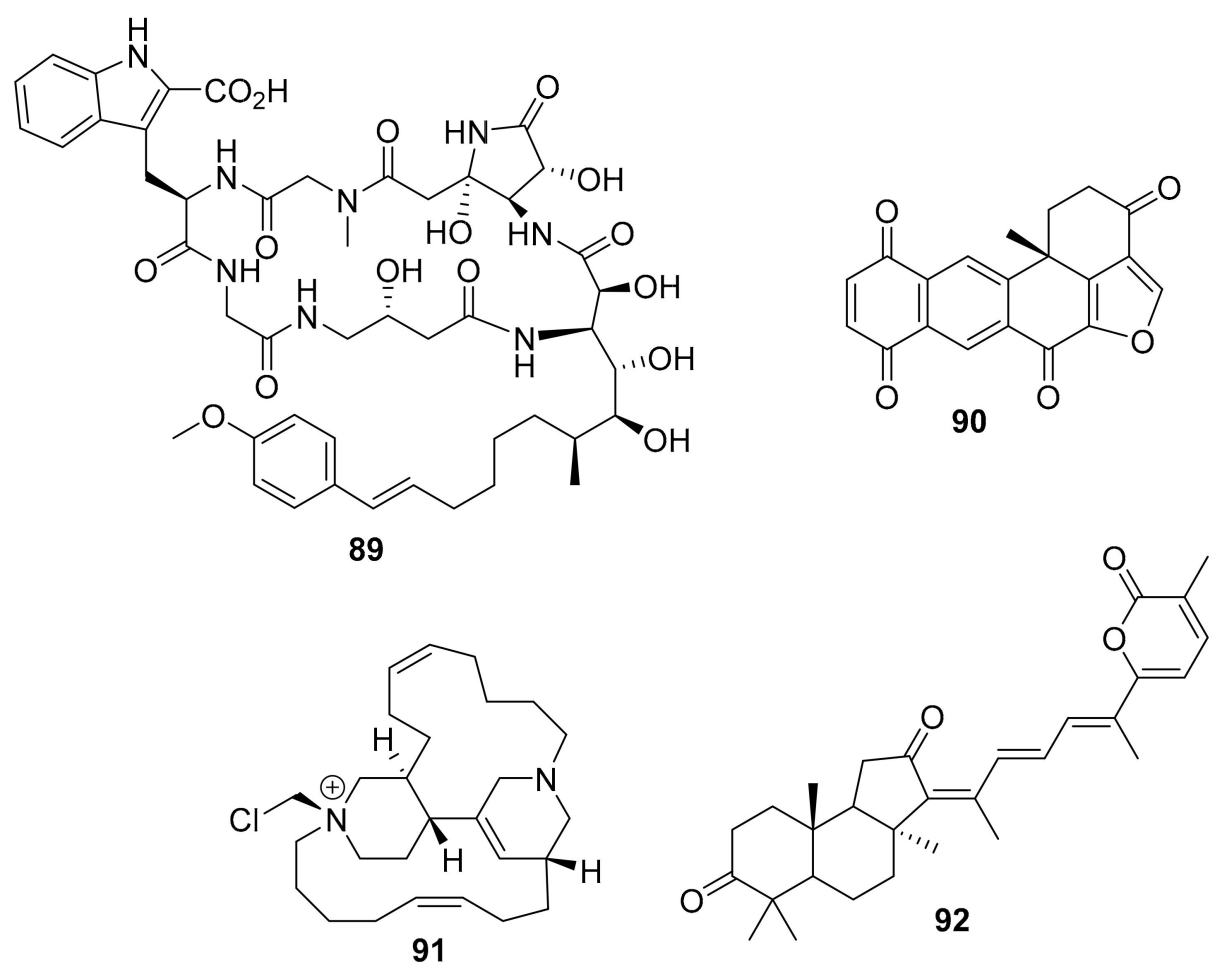
Structure of compounds **89**–**92**.

In 2015, Esther A. Guzmán et al. [109] reported microsclerodermin A (**89**, Figure 23), from the marine sponge *Amphibleptula cf. madrepora*, and this compound inhibited GSK3β and pancreatic cancer cell viability in vitro (IC_50_ = 2.4 μM). In 2015, Shou-Ping Shih et al. isolated a marine polycyclic quinone called halenaquinone (compound **90**, Figure 23) from the marine sponge *Petrosia* sp. [110]. This molecule is a broad spectrum tyrosine kinase inhibitor, as well as in vitro cytotoxin against multiple cell types (IC_50_ = 0.18 ~ 8.0 μg/mL) [110]. In 2016, Germana Esposito et al. reported chloromethylhalicyclamine B (**91**, Figure 23) after it was isolated from the sponge *Acanthostrongylophora* sp., and suggested that it “can efficiently interact with the ATP-binding site of CK1δ (IC_50_ = 6 μM) in spite of its globular structure, very different from the planar structure of known inhibitors of CK1δ [111]. As the authors also suggested, this molecule can be a lead molecule for the creation of new CK1δ/ε inhibitors. In 2016, Ran Wang et al. discovered stellettin B (**92**, Figure 23) from the marine sponge *Jaspis stellifera*, and it inhibited phosphorylation of PI3K and Akt [112]. Moreover, compound **92** exhibited potent activitivity against human glioblastoma cancer SF295 cells (IC_50_ = 0.01 μM).

**Figure 24:**
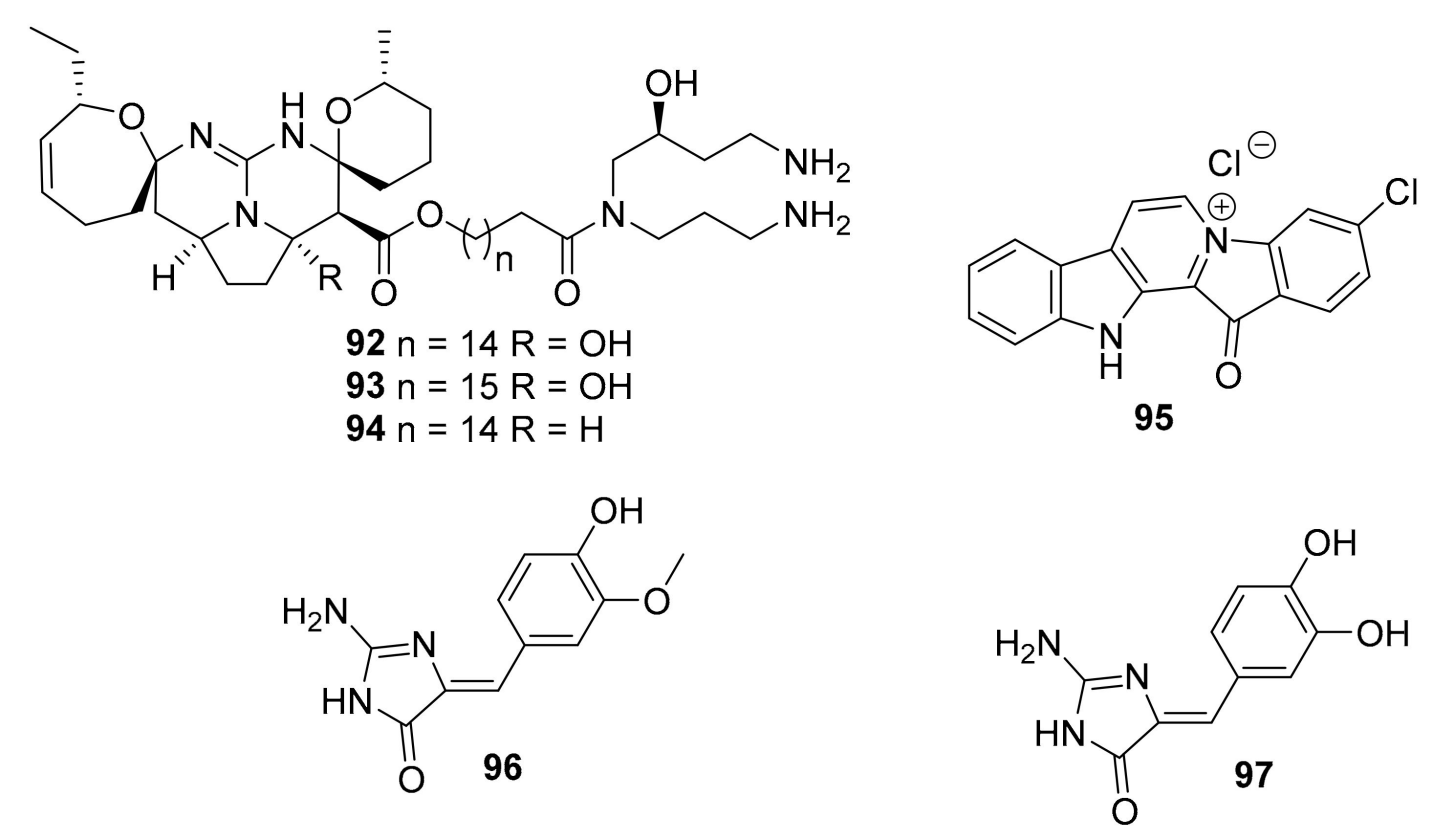
Structures of compounds **92**–**97**.

In 2016, María Roel et al. reported that crambescidins 816, 830, and 800 (compounds **93**–**95**, Figure 24) were isolated from the marine sponge *Smenospongia* sp. [113]. These compounds inhibited tumor cell proliferation by suppressing CDK 2/6 expression and activated the cell CDK inhibitors -2A, -2D and -1A, suggesting some potential as anticancer agents [113]. In 2017, Nadège Loaëc et al. reported polyandrocarpamines A and B (**96** and **97**, Figure 24) as synthetic analogues of the 2-aminoimidazolin-4-one scaffold previously isolated from marine calcareous sponges *Leucetta* and *Clathrina* [114]. Compounds **96** and **97** were found to be nM potency inhibitors of DYRKs (IC_50_ = 0.17 ~ 0.88 μM) and CLKs (IC_50_ = 0.32 ~ 8.6 μM), and the 2-aminoimidazolone scaffold was accordingly proposed as having promise for developing new kinase inhibitors [114]. 

**Figure 25:**
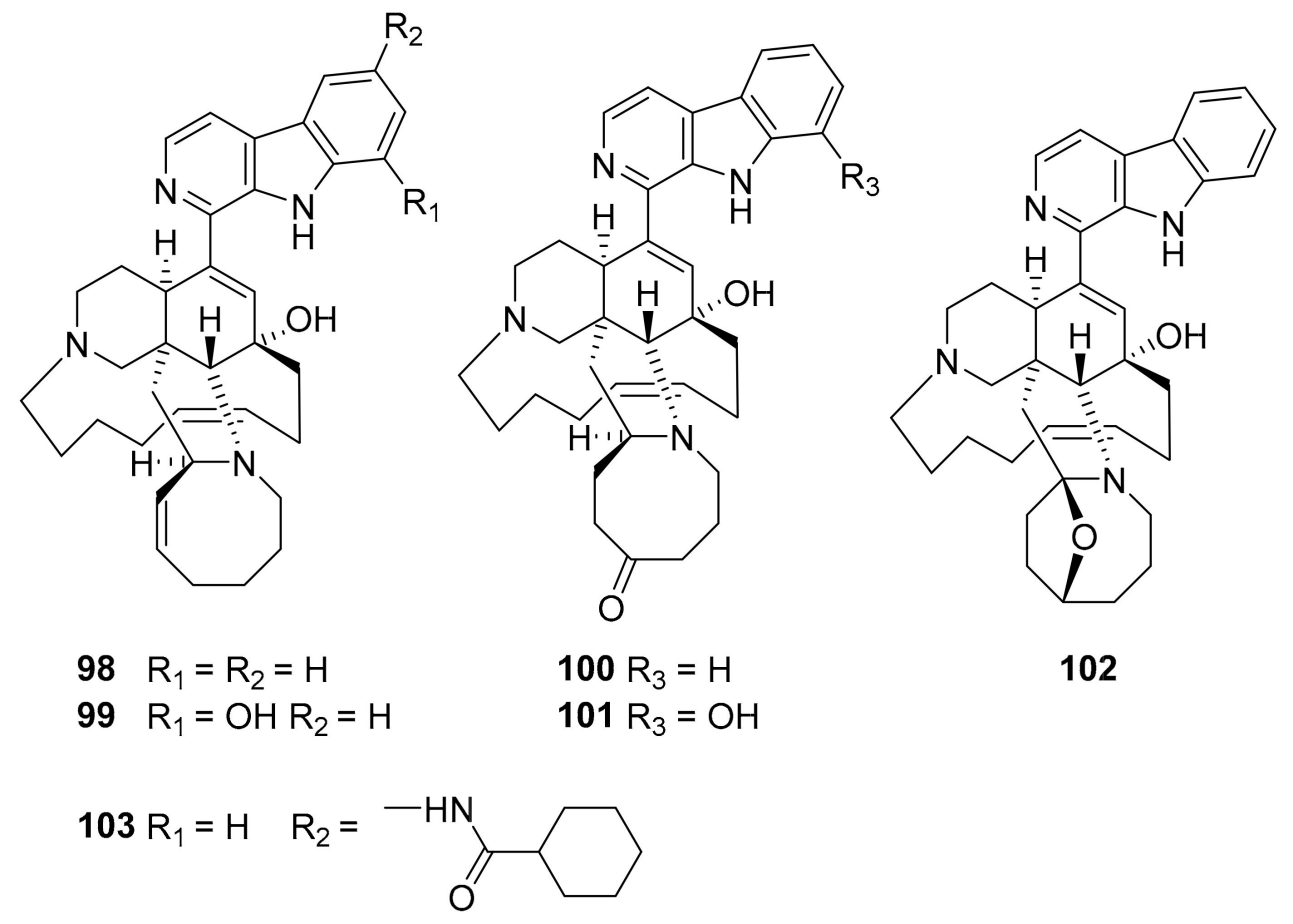
Structures of compounds **98**–**103**.

**Figure 26:**
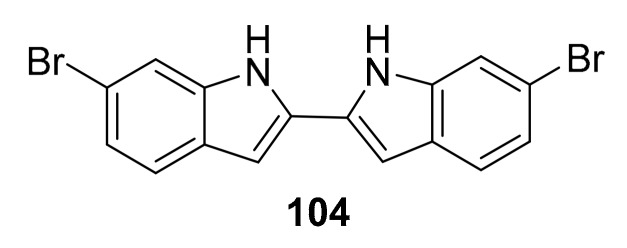
Structure of the bis-indole compound, **104**.

In 2018, the manzamine alkaloids were isolated from Indo-Pacific marine sponges *Acanthostrongylophora* sp., including manzamine A (**98**), 8-hydroxymanzamine A (**99**), manzamine E (**100**), manzamine F (**101**), 6-deoxymanzamine X (**102**), and the synthetic analogue 6-cyclohexamidomanzamine A (**103**) was also generated [115,116]. These compounds (Figure 25) showed mixed noncompetitive inhibition of MtSK, and the synthetic compound **103** takes advantage of the structural features to pursue applications of anti-inflammatory, antiparasitic, insecticidal, and antibacterial activities with potential for development against malaria and tuberculosis [115,116]. In 2014, Nag S. Kumar et al. reported the discovery of a series of bis-indole alkaloids based on the structure of a marine alkaloid isolated from the marine sponge *Topsentia pachastrelloides*, of which compound **104** (Figure 26) is a promising PK inhibitor [117].

**Scheme 4:**
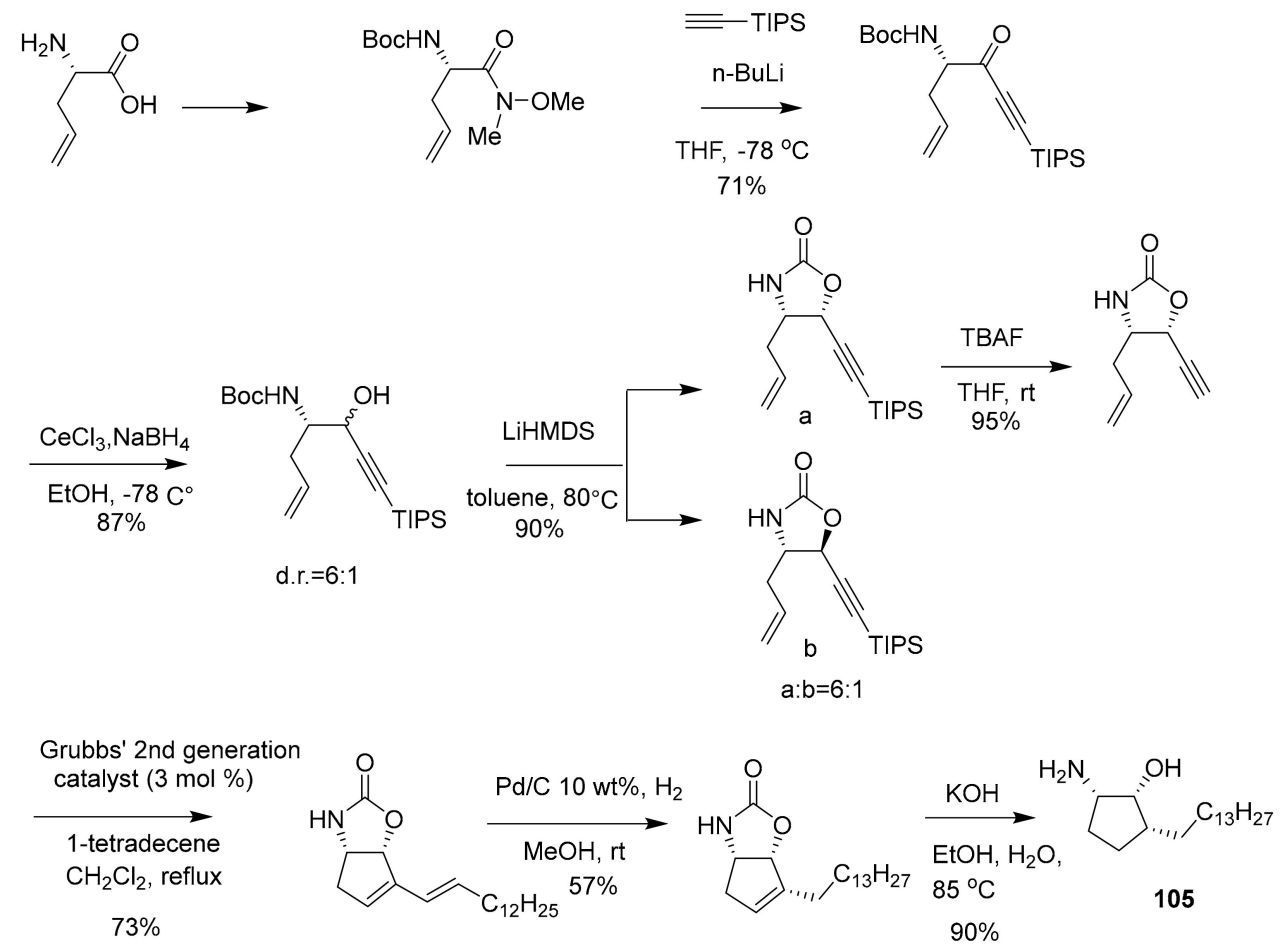
Synthetic route and structure of pachastrissamine carbocyclic analogue **105**.

**Scheme 5:**
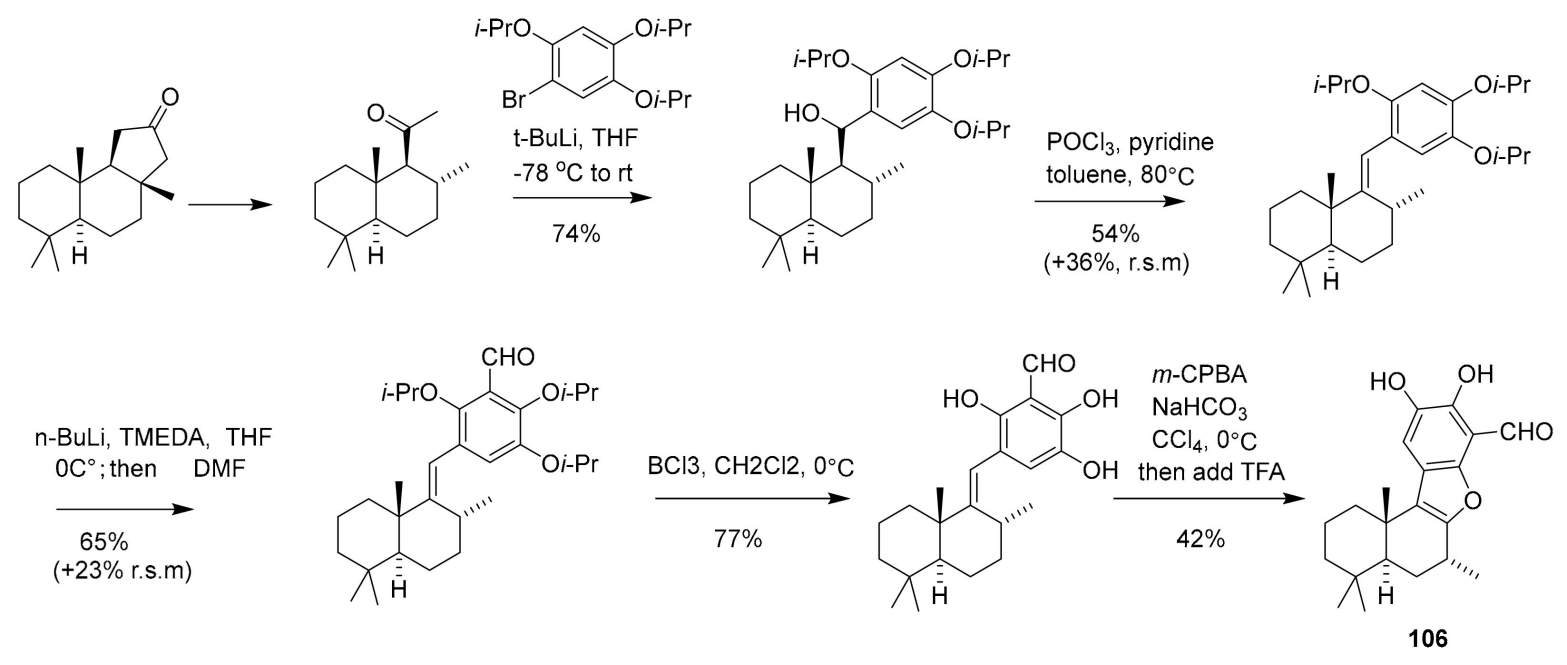
Synthesis and structure of (+)-liphagal (**106**).

In 2015, Yongseok Kwon et al. synthesized compound **105** (Scheme 4) as a synthetic analogue of pachastrissamine (first reported in 2002 from the marine sponge *Pachastrissa* sp.), and it has in vitro cytotoxicity along with sphingosine kinase inhibitory activity that was deemed superior to the natural lead compound [118,119]. The IC_50_ of compound **105** and pachastrissamine in the inhibition of SphK1 and SphK2 were 7.5 μM and 12.0 μM, 20.1 μM and 41.8 μM, respectively, suggesting that compound **105** could have preferable usage in related pharmaceutical applications [118,119]. The synthetic route of the pachastrissamine carbocyclic analogue **105** is depicted in Scheme 4. The compound (+)-liphagal (**106**, Scheme 5) was first isolated in 2006 from a marine sponge by Frederick and Andersen et al. [120] and in 2015 the chemical synthesis was completed by Markwell-Heys et al. [121]. This compound inhibited PIK-α (IC_50_ = 100 nM), and was also found to be cytotoxic to several tumor cell lines with IC_50_ ≈ 1 μM. It has been suggested that **106** has potential application as a new type of kinase inhibitor or even cancer drug [122]. The synthetic route of (+)-liphagal is depicted in Scheme 5, which initiates with (+)-sclareolide (commercially available) and generates the final product in ~10% yield after 13 reaction steps [122].

### 2.2. Preclinical and Clinical Candidates

**Figure 27:**
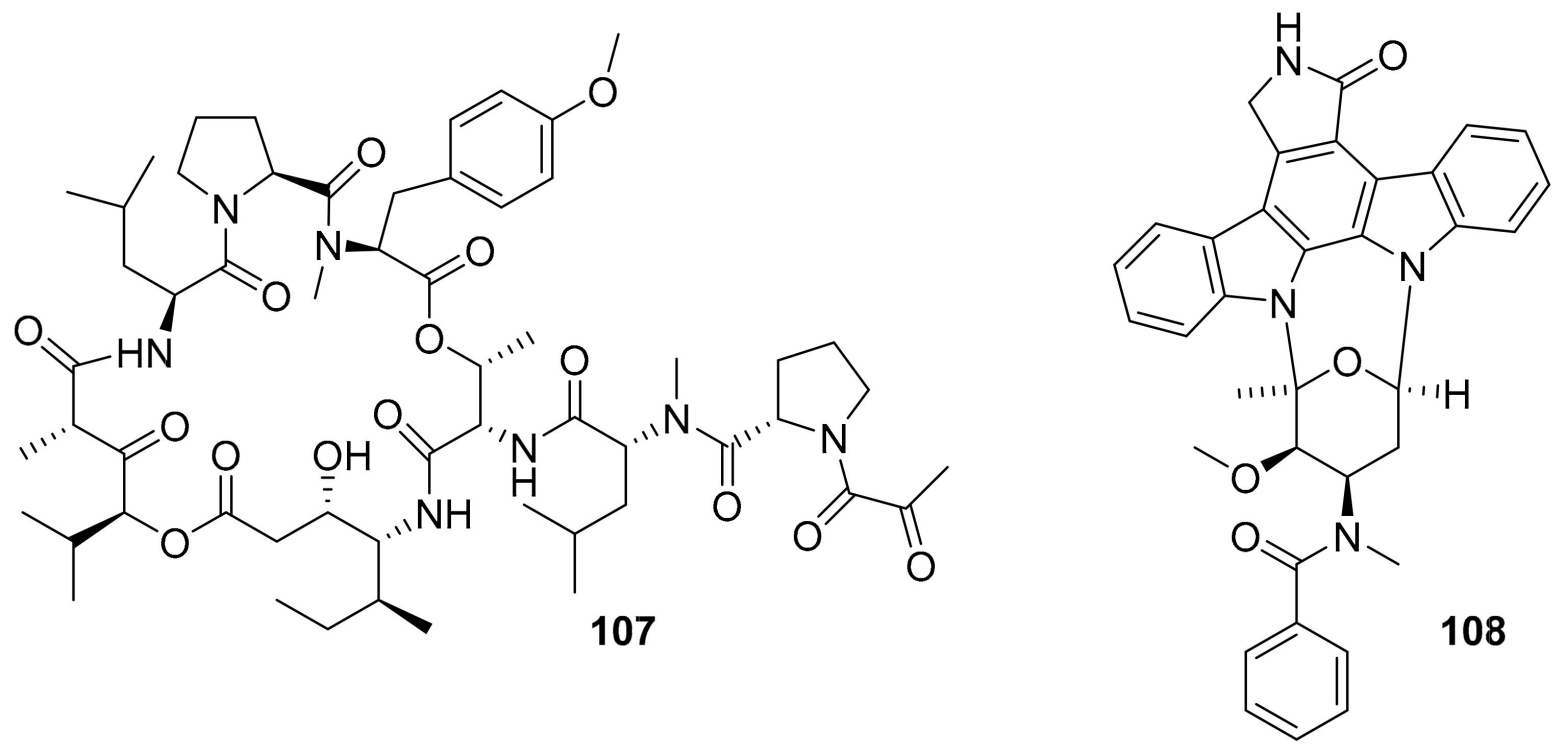
Structures of marine-derived kinase inhibitors in clinical use (**107**–**108**).

**Figure 28:**
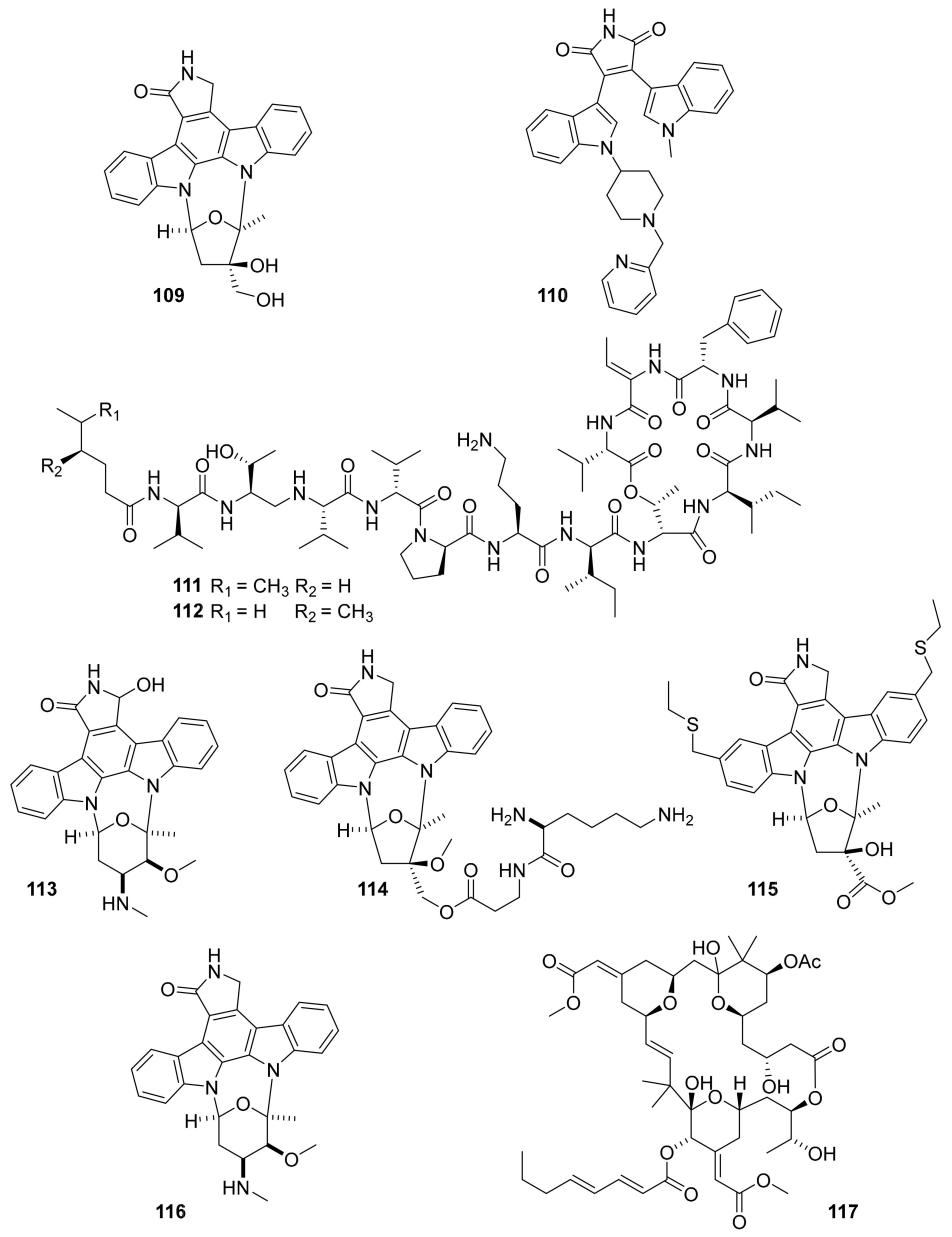
Structures of marine-derived kinase inhibitors, **109**–**117**, currently in clinical trials.

The US FDA has already approved more than 34 small molecule kinase inhibitors for human use [123]. Many of these have been used for the treatment of cancers, although toxicity is of great concern. A number of marine-derived kinase inhibitors have entered clinic trials, and some are already in later pre-approval stages or have been recently approved (Figure 27 and Figure 28 and Table 7). For example, plitidepsin (aplidin) is a marine natural product that was developed by the Spanish company PharmaMar as an inhibitor of EGPR, Src, JNK and p38 MAPK and, although there were dose-limiting toxicities in some cases, it was not fully withdrawn from clinical trials during phase II [124,125,126]. Later, it was approved for clinical use in Australia in 2018 for the treatment of relapsed/refractory multiple myeloma patients. Midostaurin is among the important drugs for aggressive systemic mastocytosis (ASM) treatment as a multi-target protein kinase inhibitor, and it was FDA-approved in 2017 [124,127,128,129]. Protein kinase inhibitors from the marine origin that have entered clinical trials include three kinase inhibitors (lestaurtinib [130], enzastaurin [131], CEP-1347 [14,124,127]) in phase III, three kinase inhibitors (kahalalide F [132,133], 7-hydroxystaurosporine [134,135], staurosporine [136]) in phase II and two kinase inhibitors (CEP-2563 [14,124,127], isokahalalide F [14,124,127]) in phase I clinical studies. Somewhat related, bryostatin 1 entered phase II clinical trials for testing in the treatment of Alzheimer’s Disease as a PKC activator, after it was also experimentally used in phase I trials for various forms of cancer [137,138,139]. It will be interesting to see the future developments of this marine natural product drug candidate and others.

## 3. Conclusions

**Figure 29:**
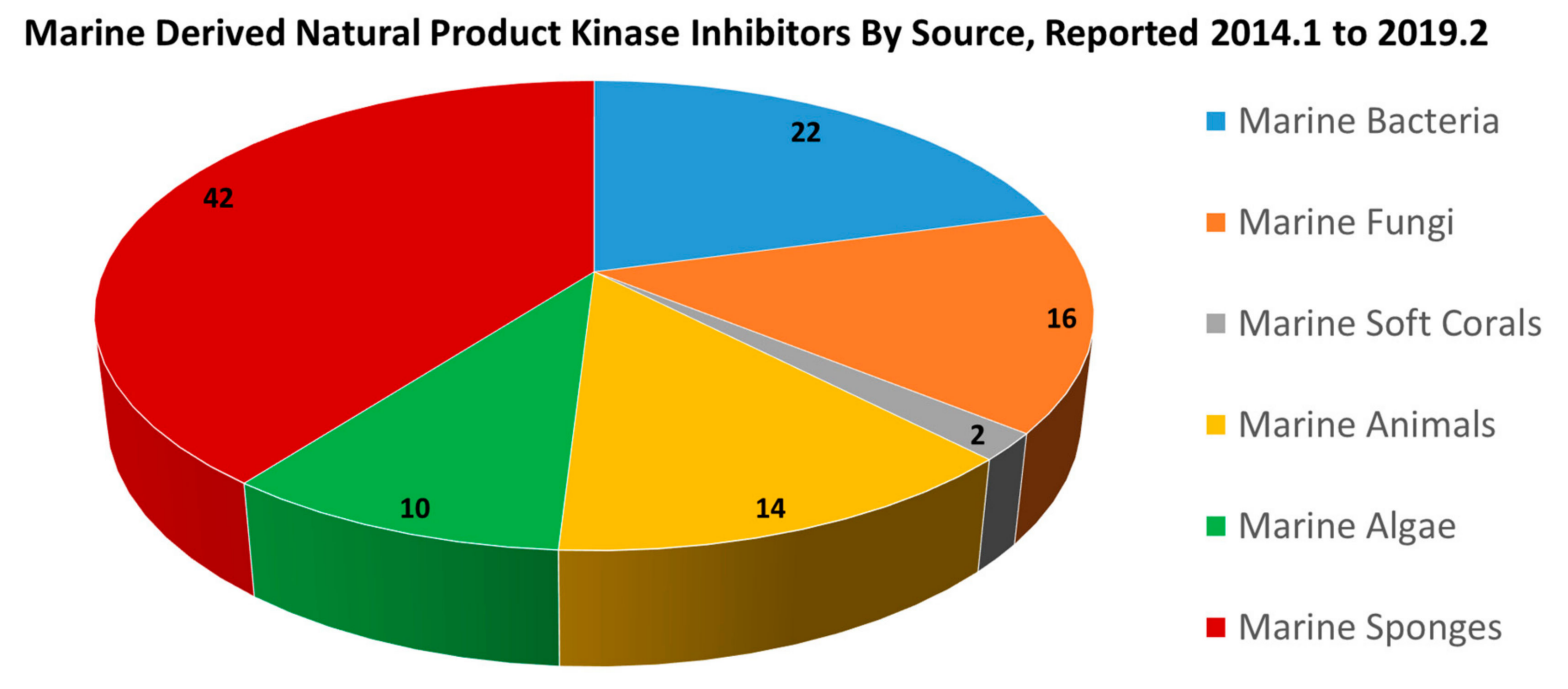
A breakdown by class of the reported producing organism for the marine derived natural product kinase inhibitors covered in this review period, from January, 2014 to February, 2019.

A wide variety of kinase inhibitors have been recently published from marine-derived sources, including bacteria and cyanobacteria, fungi, animals, algae, soft corals and sponges. More than 100 kinase inhibitors of marine origin have been systematically documented for facile examination and comparison. An evaluation of the number of compounds reported during the period of this review, separated by the class of source organism, shows that there is a vast opportunity area for a kinase inhibitor research on the natural product chemistry of marine soft corals, especially (Figure 29). However, it should also be considered that from the overwhelming 58 kinase inhibitors reported from marine animals, soft corals, and sponges during this period, many are likely produced by symbiotic microorganisms. It is equally important to realize, however, that those symbionts may be difficult or impossible to cultivate in laboratory conditions. It is thus of increasing importance that the development of selective kinase inhibitors from marine natural product drug discovery leads has utilized active fragment (pharmacophore) based medicinal chemistry to overcome supply limitations of natural product drug leads, and further optimize the drug-like properties of these molecules. In this way, each newly reported compound may provide marine-derived scaffolds and bioactivity information for drug screening, and many have been found to have significant biological properties that could be used in the treatment of different conditions such as cancers, inflammation, and neurodegenerative diseases, for example.

The noted structural differences and diversity of the marine natural products and their derivatives are important for chemical properties, conferring pharmacological effects, and of late-stage interest, intellectual property management. Some recent marine drug approvals include plitidepsin (aplidin), an ascidian natural product, and midostaurin, which is a synthetic derivative of the marine and terrestrial microbial molecule, staurosporine. There is also a pipeline of clinical trial candidates and preclinical development projects behind these drugs.

In recent years, new techniques including deep sea sampling, advanced methods in chemical synthesis, more efficient target-based isolation [140], along with computational database mining strategies and high-throughput screenings have been widely helpful for drug candidate discovery and design. These all improve the efficiency and success rate of the drug development process. Since kinases are directly implicated in so many aspects of disease development and progression in growth, successful targeting of these proteins has resulted in a number of novel drugs for cancer and neurodegenerative diseases and is expected to continue to do so. Nevertheless, while most of the approved kinase inhibitors are ATP binding site competitors, there is a dire need to overcome the toxicity associated with this liability. Hence, marine-derived natural product kinase inhibitors could serve as lead compounds for development by medicinal chemistry. These diverse compounds greatly improve the chemical space covered during screening for new kinase inhibitors, with the goal of eventually providing new clinical candidates for unmet medical needs.

## Figures and Tables

**Table 1:** A summary of kinase inhibitors isolated from marine bacteria, 2014–2019.

Compound	Chemical Class	Marine Source	Drug Targets/Inhibitory Activity	References
**1**	lipopeptide	*Bacillus megaterium*	p-Akt, p-MAPK, p-GSK-3β	[33]
**2** **3–5**	indolocarbazole alkaloids	*Streptomyces* sp. A65	inactive analogue PKC, BTK (0.25–1.91 μM)	[34]
**6–8**	indolocabazole derivatives	*Streptomyces* sp. A68	PKC-α, BTK, ROCK2 (0.91–1.84 μM)	[35]
**9–12**	cyclizidine alkaloid	*Streptomyces* sp. HNA39	ROCK2 (7.0–42 μM)	[36,37,38,39]
**13**	Indolocarbazole alkaloid indolocarbazole alkaloid indolocarbazole alkaloid	*Streptomyces* sp. DT-A61	ROCK2 (5.7 nM)	[40]
**14**		*Streptomyces* sp. DT-A61	PKC-α (92 nM)	[40]
**15**		*Streptomyces* sp. A22	PKC, BTK, ROCK2 (0.91–1.84 μM)	[41]
**16** **17–21**	staurosporine derivatives	*Streptomyces* sp. NB-A13	inactive analogue PKC-θ (0.06–9.43 μM)	[42]
**22**	naphthoquinone	*Streptomyces* sp. XMA39	ROCK2 (>20 μM)	[43]

**Table 2:** A summary of kinase inhibitors isolated from marine fungi, 2014–2019.

Compound	Chemical Class	Marine Source	Drug Targets/Inhibitory Activity	References
**23**	isochromen-6-one	*Penicillium* sp. ZJ27	PKnG (76.5 μM)	[41]
**24**	alkaloid	*Penicillium* sp.	p-p38 MAPK	[45]
**25**	polyketide	*Diaporthe* sp. HLY-1	IKK	[46]
**26**	indole diterpenoid	*Aspergillus flavus* OUCMDZ-2205.	PKC-β (15.6 μM)	[47]
**27**	furan derivative	*Xylaria* sp. (No. 2508)	p-Akt, p-ERK1/2	[48]
**28**	dihydro-isocoumarin	*Aspergillus* sp. SF-5976	p-p38 MAPK	[49]
**29**	phenylspiro-drimane derivative	*Stachybotrys* sp. KCB13F013	p-ERK, p-JNK, p-p38 MAPK	[50]
**30**	benzocoumarin	*Aspergillus* sp. LF660	GSK-3β (0.35 ± 0.04 μM)	[51]
**31**			GSK-3β (0.13 ± 0.04 μM)	
**32**			GSK-3β (0.20 ± 0.04 μM)	
**33**	lipophilic depsipeptide	*Alternaria* sp. SF-5016	p-JNK, p-p38 MAPK	[52]
**34**	citreohybridonol	*Toxicocladosporium* sp. SF-5699	p-p38 MAPK	[53]
**35**	2-methyl-hydroquinone	*Penicillium* sp. HL-85-ALS5-R004	p-Akt, p-ERK1/2	[54]
**36**	isopyrrolo-naphthoquinone	*Biscogniauxia mediterranea*	GSK-3β (8.04 μM)	[55]
**37**	polyketides	*Penicillium* sp. SF-5629	p-p38 MAPK	[56]
**38**		*Oidiodendron griseum* UBOCC-A-114129	CLK1 (15.6 g/mL)	[58]

**Table 3:** A summary of kinase inhibitors isolated from marine soft coral, 2014–2019.

Compound	Chemical Class	Marine Source	Drug Targets/Inhibitory Activity	References
**39**	simple sulfone alcohol	*Cladiella australis*	MAPK, AKt	[59]
**40**	eunicellin diterpenoid	*Cladiella pachyclados*	EGFR	[60]

**Table 4:** A summary of kinase inhibitors isolated from marine animals, 2014–2019.

Compound	Chemical Class	Marine Source	Drug Targets/Inhibitory Activity	References
**41–42**	spiroketals	marine ascidian genus *Didemnum*	CDK5, CK1, DyrK1A, GSK-3 (10 μg/mL)	[62]
**43–52**	purine alkaloids	marine tunicate *Symplegma rubra*	CDK5, CK1, DyrK1A, GSK-3 (10 μg/mL)	[63]
**53**	saponin sulfate	sea cucumber	PAK1 (1.2 μM) LIMK (60 μM) AKT (59 μM)	[64,65]
**54**	anthraquinone derivative	marine echinoderm *Comanthus* sp.	IGF1-R (5 μM) FAK (8.4 μM) EGFR (4 μM)	[66]

**Table 5:** A summary of kinase inhibitors isolated from marine algae, 2014–2019.

Compound	Chemical Class	Marine Source	Drug Targets/Inhibitory Activity	References
**55**	bromophenol	red alga *Rhodomelaceae confervoides.*	RTKs, p-PKB/Akt	[68,69]
**56**	phlorotannin	brown seaweed *Eisenia bicyclis*	p-AKt	[70]
**57**	carotenoid	brown alga wakame seaweeds wakame (*Undaria pinnatifida*) and kombu (*Laminaria japonica*)	AKt, MAPK	[71,72,73]
**58**	bromophenol	red alga *Polysiphonia morrowii*	p-ERK	[74]
**59–60**	lipopoly-saccharide	brown seaweed marine alga (*Eisenia bicyclis*)	p-ERK1/2, p-JNK	[75]
**61^a^**	cyclic depsipeptide	marine cyanobacterium *Moorea producens* (formerly *Lyngbya majuscula*)	RTKs	[76]
**62 ^a^**	bromo-honaucin	marine cyanobacterium *Leptolyngbya crossbyana*	AKt, ERK	[77,78]
**63 ^b^**	cyclic depsipeptide	marine cyanobacterium *Moorea producens*	JNK	[79,80]
**64**	cyclic depsipeptide	marine cyanobacterium *Leptolyngbya* sp.	VEGFA/VEGFR2	[81,82]

**^a^** This molecule is synthetic but designed based on a marine natural product. **^b^** Hoiamide A is JNK activator.

**Table 6:** A summary of kinase inhibitors isolated from marine sponges, 2014–2019.

Compound	Chemical Class	Marine Source	Drug Targets/Inhibitory Activity	References
**65**	bromopyrrole alkaloid	*Callyspongia* sp. (CMB-01152).	CK1, CK5, GSK3β	[84]
**66**	indole alkaloids	*Hyrtios erecta*	STK (24.5 μg/mL)	[88]
**67**			STK (13.6 μg/mL)	
**68**			STK (18.6 μg/mL)	
**69**	sipholane triterpenoid	*Callyspongia siphonella.*	p-Brk, p-FAK	[89]
**70**	indole alkaloid	*Geodia barretti*	RIPK2 (8.0 μM) CAMK1a (5.7 μM) SIK2 (6.1 μM)	[90,92,93]
**71**	diarylpyrazine	*Hamacanthins*	PDGF-Rβ (0.02 μM)	[93]
**72**	tryptoline analogues	*Fascaplysinopsis* sp.	CDK4/D1 (0.35 μM)	[94,95,96,97,98]
**73 ^a^**			CDK4 (10 μM)	
**74 ^a^**	alkaloid derivative	*Fascaplysinopsis* sp.	PI3K/Akt	[99]
**75**	bisindole alkaloids	*Topsentia pachastrelloides*	MRSA PK (60 nM)	[100]
**76**			MRSA PK (16 nM)	
**77**			MRSA PK (1.4 nM)	
**78**	macrocyclic oxaquinolizidine alkaloid	*Xestospongia* species.	PI3K, Akt	[101]
**79–84**	bromopyrrole alkaloids	*Stylissa massa* and *Stylissa flabelliformis*	GSK-3, DYRK1A, CK-1 (0.6–6.4 μM)	[102]
**85**	adociaquinone derivatives	*Xestospongia* sp.	inactive analogue	[108]
**86**			CDK9/cyclin T (3 μM)	
**87–88**			CDK5/p25 (6 μM)	
**89**	cyclic peptide	*Amphibleptula*	GSK-3β	[109]
**90**	polycyclic quinone	*Petrosia*	hexokinase II	[110]
**91**	halogenated alkaloid	*Acanthostrongylophora ingens*	CK1δ/ε (6 μM)	[111]
**92**	isomalabaricane triterpenoid	*Jaspis stellifera*	PI3K, Akt	[112]
**93–95**	guanidine alkaloids	*Smenospongia* sp.	CDK2/6	[113]
**96–97**	2-aminoimidazolone alkaloids	*Leucetta* and *Clathrina*	DYRKs (0.17 ~ 0.88 μM) CLKs (0.32 ~ 8.6 μM)	[114]
**98–103**	manzamine alkaloids	*Acanthostrongylophora* sp.	MtSK	[115,116]
**104**	bis-indole alkaloid	*Topsentia pach astrelloides*	MRSA PK	[117]
**105**	sphingolipid	*Pachastrissa* sp.	SphK1 (7.5 μM) SphK1 (20.1 μM)	[118,119]
**106**	polyfused-benzofuran	*Aka coralliphaga*	PIK-α (100 nM)	[122]

^a^ This molecule is synthetic but designed based on a marine natural product.

**Table 7:** Preclinical and clinical status of some kinase inhibitors, and bryostatin 1, with marine-derived natural product origins ^a,b^.

Compound Name (Trademark)	Target	Marine Source	Chemical Class	Company or Institution	Therapeutic Area	Status	References
plitidepsin (aplidin, **107**)	VEGFR1	ascidian	depsipeptide	PharmaMar	multiple myeloma	FDA-approved (2018)	[124,125,126,127]
midostaurin (**108**)	PKC-α, KIT, FLT3, VEGFR2, PDGFR-β	ascidian / bacteria	indolocarbazole	Novartis	acute myeloid leukemia,	FDA-approved (2017)	[124,127,128]
lestaurtinib (CEP-701, **109**)	Flt-3, JAK-2	ascidian / bacteria	indolocarbazole	Kyowa kirin	acute lymphoblast leukemia	Phase III	[127,130]
enzastaurin (LY317615, **110**)	PKCβ, Class I PI3K	ascidian / bacteria	indolocarbazole	Eli Lilly	diffuse large B cell lymphoma	Phase III	[127,131]
kahalalide F (PM-92102, **111**)	EGFR	mollusk and algae	tridecapeptide	PharmaMar Cephalon	cancar	Phase II (terminated)	[127,132,133]
isokahalalide F (PM02734, elisidepsin, **112**)	ErbB2	mollusk and algae	tridecapeptide	PharmaMar	cancer	Phase I (terminated)	[14,124,127]
7-hydroxystaurosporine (UCN-01, **113**)	Chk2, PDPK1, Chk1 PKC	ascidian / bacteria	indolocarbazole	Kyowa kirin	cancer	Phase II (terminated)	[127,134,135,]
CEP-2563 (**114**, prodrug of CEP-751)	Trk-A, Trk-B, Trk-C	ascidian / bacteria	indolocarbazole	Cephalon	cancer	Phase I (terminated)	[14,124,127]
CEP-1347 (KT7515; **115**)	MAP3K11	ascidian	indolocarbazole	Teva	Parkinson’s	Phase III (terminated)	[14,124,127]
staurosporine (AM-2282, **116**)	PKC, JAK2, CamKIII	ascidian / bacteria	indolocarbazole	Kyowa Hakko Kirin (originator)	multiple myeloma	Phase II (terminated)	[127,136]
bryostatin 1 *^b^* (**117**)	PKC	bryozoan	macrocytic lactone	Neurotrope Bioscience	Alzheimer’s	Phase II (terminated)	[127,137,138,139]

^a^ Staurosporine and several derivatives are included here. These compounds have been derived from terrestrial soil bacteria, ascidians and marine bacteria. ^b^ bryostatin 1 is a Protein Kinase C (PKC) activator.

## List of Abbreviations

AKt	Serine/ threonine kinase
BTK	Brution tyrosine kinase
BrK	Breast tumor kinase
CLK1	Cdc2-like kinase 1
CLKs	Cdc2-like kinases
CDK1	Cyclin-dependent kinase 1
CDK2	Cyclin-dependent kinase 2
CDK4	Cyclin-dependent kinase 4
CDK5	Cyclin-dependent kinase 5
CDK6	Cyclin-dependent kinase 6
CDK9	Cyclin-dependent kinase 9
CK1	Casein kinase 1
CK5	Casein kinase 5
CAMK1a	Calcium/calmodulin-dependent protein kinase 1a
CDKN1C	Cyclin-dependent kinase inhibitor 1C
CDKN2B	Cyclin-dependent kinase inhibitor 2B
CHEK2	Checkpoint kinase 2
Class I PI3K	PI3-kinase class I
Chk	Csk homologous kinase
CaMKII	Calmodulin-dependent protein kinase II
DYRK1A	Dual-specificity tyrosine phosphorylation regulated kinase-1A
DYRKS	Dual-specificity tyrosine phosphorylation regulated kinases
ERK	Extracellular signal-regulated kinase
ERK1/2	Extracellular signal-regulated kinase1/2
EGFR	Epidermal growth factor receptor
FAK	Focal adhesion kinase
FGER	Fibroblast Growth Factor Receptor
GSK-3	Glycogen synthase kinase 3
GSK-3β	Glycogen synthase kinase 3 beta
IKK	IkappaB kinase
IGF1-R	Insulin-like growth factor 1 receptor
JNK	C-Jun NH-terminal kinase
LIMK	LIM kinase
MtSK	Shikimate kinase from mycobacterium tuberculosis
MRSA PK	MRSA pyruvate kinase
MAPK	Mitogen-activated protein kinases
MAP3K11	Mitogen-activated protein kinase kinase kinase11
MIC	Minimum inhibitory concentration
NF-κB	Nuclear factor – kappa B
PKC-α	Protein kinase C alpha
PKC-β	Protein kinase C beta
PKnG	Protein kinase G
PKB	Protein kinase B
p-AKt	Phosphorylated AKt
p-ERK1/2	Phosphorylated extracellular signal-regulated kinase
PAK1	p21-activated kinase 1
PI3K	Phosphatidylinositol 3-kinase
PDGFR-α	Platelet-derived growth factor receptor alpha
PDGFR-β	Platelet-derived growth factor receptor beta
PDPK1	Phosphatidylinositide-dependent protein kinase 1
ROCK2	Rho-associated protein kinase
RTKs	Receptor tyrosine kinase
RIPK2	Receptor-interacting serine/threonine kinase 2
STK	Serine/threonine kinase family
SIK2	Salt-inducible kinase2
SphKs	Sphingosine kinases
STAT3	Signal transducer and activator of transcription 3
VEGFA	Vascular endothelial growth factor A
VEGFR2	Vascular endothelial growth factor receptor 2
JAK2	Tyrosine-protein kinase JAK2
